# Enhanced Pathogenic Consequences Induced by a Seven-Amino-Acid Extension in the G Protein of the HRSV BA9 Genotype

**DOI:** 10.3390/ijms26052081

**Published:** 2025-02-27

**Authors:** Na Wang, Jingjing Song, Lei Cao, Naiying Mao, Yuqing Shi, Jie Jiang, Wuyang Zhu, Yan Zhang

**Affiliations:** 1 National Key Laboratory of Intelligent Tracking and Forecasting for Infectious Diseases, National Institute for Viral Disease Control and Prevention, Chinese Center for Disease Control and Prevention, Beijing 102206, China; 2 NHC Key Laboratory of Medical Virology and Viral Disease, National Institute for Viral Disease Control and Prevention, Chinese Center for Disease Control and Prevention, Beijing 102206, China

**Keywords:** attachment protein (G), respiratory syncytial virus (RSV), reverse genetics, virulence

## Abstract

In a previous outbreak of the human respiratory syncytial virus (HRSV), we identified a variant strain of genotype BA9 with a seven-amino-acid extension (Q-R-L-Q-S-Y-A) at the C-terminus of the attachment protein (G). To assess the impact of this extension on the virulence of HRSV, two full-length infectious clones using the wild strain of genotype BA9 as a backbone, one containing the seven-amino-acid extension (rRSV BA9 WT), and the other deleting this extension (rRSV BA9 Δ7AA), were successfully rescued using a reverse genetics system. The biological properties and virulence of the two rescued viruses were then compared and analyzed in vitro and in vivo. Compared to the rRSV BA9 Δ7AA, the rRSV BA9 WT exhibited a larger plaque size and a more pronounced suppression of the host cell innate immune response in vitro (IFN-β levels: 154.33 pg/mL vs. 11.27 pg/mL). The rRSV BA9 WT demonstrated increased adaptability in mice, with a 10-fold higher lung viral load and a stronger inflammatory response following intranasal exposure. Our study primarily demonstrated that the C-terminal extension of the G protein of the HRSV can enhance viral virulence, underscoring the importance of virological surveillance in the prevention and treatment of severe HRSV-related disease.

## 1. Introduction

The human respiratory syncytial virus (HRSV) is an enveloped, negative-sense, single-stranded RNA virus belonging to the family of *Pneumovirus* and the genus *Orthopneumovirus* [1]. The HRSV is a major pathogen responsible for respiratory infections in both the upper and lower respiratory tract, causing clinical manifestations such as bronchiolitis and pneumonia. Children, the elderly, and immunocompromised individuals are most vulnerable to HRSV-associated lower respiratory tract infections (LRTIs). Globally, the HRSV is estimated to cause approximately 33 million infections annually in children under the age of five, resulting in more than 100,000 deaths [2].

HRSV strains are categorized into two subtypes, HRSV-A and HRSV-B, based on the diversity of the attachment protein (G). In recent years, a 72-nucleotide duplication in the G protein of genotype ON1 (subgroup A) and a 60-nucleotide duplication in genotype BA (subgroup B) have emerged as the predominant strains on a worldwide scale. These genetic variations have been associated with the increased global prevalence of their respective genotypes [3]. The 60-nucleotide duplication in the BA genotype has been shown to enhance its adsorption capacity, contributing to its epidemiological success [4].

In our previous study, an outbreak of genotype BA9 in a maternity center in northern China led to 7 of 16 hospitalized neonates being admitted to the intensive care unit (ICU) in the 2021 winter. A variant strain of genotype BA9 was identified from this outbreak, and a seven-amino-acid extension (Q-R-L-Q-S-Y-A) was found at the G protein’s C-terminus due to a stop codon mutation, increasing its length from 311 to 318 amino acids [5]. The genotype BA9 G-extended variant was also detected in U.S.A. surveillance during 2016–2017 [6]. More recently, a study reported that this G-extended variant was associated with a higher ICU admission rate (44.1%) among children compared to the non-extended BA9 strain (25.2%) [7].

The G protein facilitates cell surface binding and promotes cellular infection, in addition to modulating the host responses that influence both immunity and disease [8]. The extension area is located within the mucin-like variable region II of G proteins’ extracellular C-terminus. To date, the impact of this G-extended variant of the HRSV remains unexplored. This present study employs reverse genetics to generate infectious complementary deoxyribonucleic acid (cDNA) clones of the HRSV, with the aim of elucidating the influence of extended G protein termini on HRSV virulence. The findings reveal that the addition of extended G protein termini significantly increases HRSV virulence, enhancing the virus’s adaptability in both in vitro and in vivo mouse models.

## 2. Results

### 2.1. Construction and Identification of rRSVs

**Figure 1:**
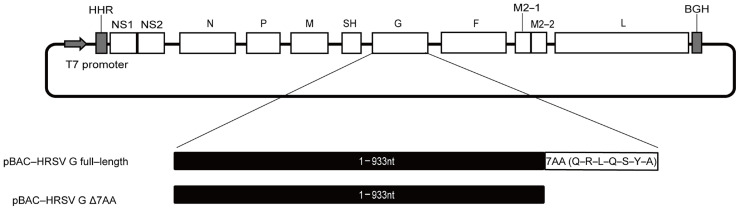
Assembly of human respiratory syncytial virus (HRSV) infectious clones, pBAC-HRSV G full-length and pBAC-HRSV GΔ7AA. Strategy for HRSV full-length complementary deoxyribonucleic acid (cDNA) clone fragments in BAC. The genome structure of HRSV relevant sequences is indicated. Abbreviations: HHR, hammerhead ribozyme; BGH, bovine growth hormone termination.

In the present study, purified RNA of HRSV/SY/2021 was extracted from pharyngeal swabs obtained from a pediatric ICU patient, suffering from respiratory and cardiac failure [5]. The complete genomic sequence of HRSV/SY/2021 is 15,242 nucleotides in length and comprises 11 gene sequences (NS1-NS2-N-P-M-SH-G-F-M2-1-M2-2-L). Sequence analysis revealed that the G gene is 954 nucleotides in length, featuring an extension of seven amino acids (“Q-R-L-Q-S-Y-A”) at the C-terminal end (Figure 1). To evaluate the impact of this seven-amino-acid extension in the G protein on HRSV virulence, two infectious clones were constructed: pBAC-HRSV G full-length (wild type) and pBAC-HRSV GΔ7AA (without the seven-amino-acid extension).

**Figure 2:**
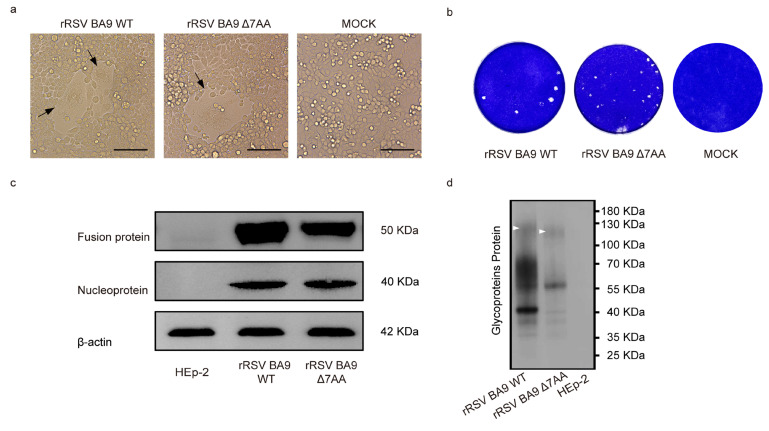
Characterization of rRSVs. (**a**) Bright-field images of the BSR-T7/9 cells infected with different viruses. Cytopathic effects (CPE) were observed at 72 h post-transfection. Representative images of mock-infected and transfected cells are shown. Black arrows: CPE caused by rRSV. Scale bars: 200 μm; (**b**) cell plaque assay of HEp-2 cells infected with rRSV BA9 WT or rRSV BA9 Δ7AA. Plaques were stained with crystal violet; (**c**) Western blot analysis of F/N protein expression of rRSV BA9 WT and rRSV BA9 Δ7AA. β-actin was used as a loading control; (**d**) Western blot analysis of G protein expression of rRSV BA9 WT and rRSV BA9 Δ7AA. White arrowheads: Mature G proteins.

To facilitate the recovery of rRSVs, infectious clones containing HRSV antigenomic cDNA were co-transfected with four plasmids encoding helper proteins into BSR/T7-9 cells. The development of cytopathic effects was monitored until clearly observable (Figure 2a). Subsequently, the recovered rRSVs were passaged blindly in HEp-2 cells. Next-generation sequencing confirmed that both rRSVs contain the correct sequences (Supplementary Materials). The viruses rescued from the pBAC-HRSV G full-length construct were designated as rRSV BA9 WT, while those derived from the pBAC-HRSV GΔ7AA construct were designated as rRSV BA9 Δ7AA for further studies. As demonstrated in Figure 2b, the plaque assay revealed that the average plaque size formed by rRSV BA9 WT (0.04 mm^2^) was larger than those produced by rRSV BA9 Δ7AA (0.02 mm^2^). Subsequently, viral protein expression was assessed via Western blot analysis using cell lysates from cells infected with either rRSV BA9 WT or rRSV BA9 Δ7AA, employing antibodies against the viral fusion protein, nucleoprotein, and β-actin as a loading control. When equivalent sample amounts were loaded, the expression level of the fusion protein in rRSV BA9 WT was marginally higher than that observed in rRSV BA9 Δ7AA (Figure 2c). Additionally, rRSV BA9 WT’s G proteins exhibited notable molecular weight variations, as confirmed by the marker labels (Figure 2d).

The amino acid extension in the variant encodes seven amino acids, “Q-R-L-Q-S-Y-A”, corresponding to glutamine, arginine, leucine, glutamine, serine, tyrosine, and alanine. Serine is a common site for O-glycosylation. O-glycosylation prediction indicated that the serine residues at positions 307 and 309 in the G-extended virus became positive O-glycosylation prediction sites compared to the non-extended virus.

These findings indicate that both recombinant viruses were successfully rescued. Furthermore, the extension of seven amino acids in the G protein significantly impacted the HRSV phenotype, leading to enhanced expression levels of the fusion protein and an increased molecular weight of the G protein.

### 2.2. Phenotypic Characterization of Recombinant Viruses In Vitro

**Figure 3:**
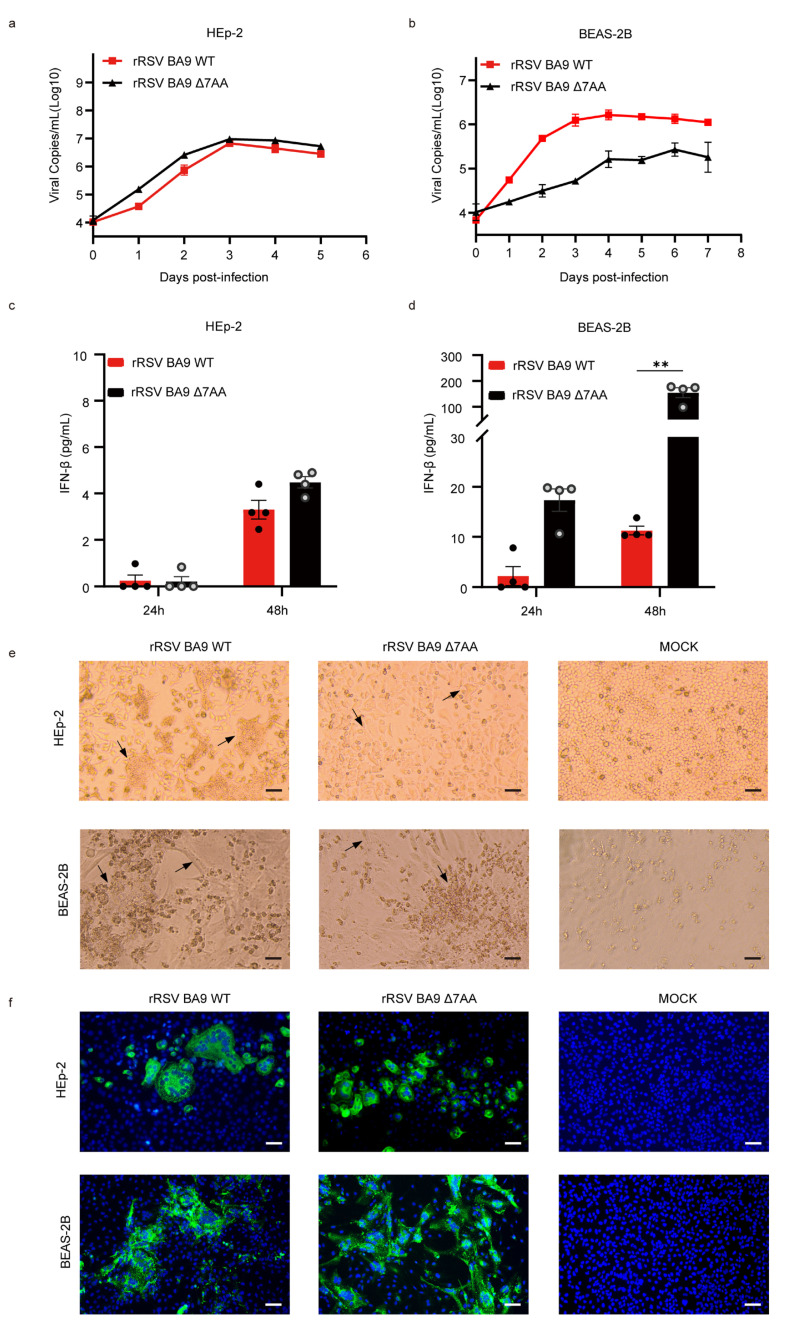
Biological characteristics of rRSV BA9 WT and rRSV BA9 Δ7AA in vitro. (**a**,**b**) Growth curves of rRSV BA9 WT and rRSV BA9 Δ7AA virus in HEp-2 or BEAS-2B cells. Determination of viral titers collected at 0 to 5 or 7 d.p.i. Data are expressed as the mean ± SEM; (**c**,**d**) IFN-β concentrations were measured by using ELISA at 24 or 48 h. **: *p*-value < 0.05; (**e**) bright-field images of the HEp-2 or BEAS-2B cells infected with different viruses. Representative images of mock-infected and transfected cells are shown. Black arrows: cytopathic effect caused by rRSV. Scale bars: 200 μm; (**f**) fluorescence analysis of rRSV-infected cells. Cells were fixed and immune-stained with a polyclonal antibody against the RSV F protein 48 hpi. Goat anti-human IgG H&L (Alexa Fluor^®^ 488, green) secondary antibodies were used, and nuclei were visualized with 4,6-diamidino-2-phenylindole. Scale bars represent 200 μm.

To assess the replication kinetics of the recombinant viruses, the growth kinetics were evaluated using rRSVs (rRSV BA9 WT and rRSV BA9 Δ7AA) at various time points post-infection in HEp-2 and BEAS-2B cells. In HEp-2 cells, both rRSV BA9 WT and rRSV BA9 Δ7AA exhibited similar replication kinetics, with viral loads increasing by day 1 and plateauing by day 3 post-inoculation. rRSV BA9 WT reached 1.27 × 10^6^ cp/mL, while rRSV BA9 Δ7AA reached 2.60 × 10^6^ cp/mL (Figure 3a). These results indicate that all recombinant viruses (regardless of the G gene extension) exhibit similar replication kinetics in HEp-2 cells. In BEAS-2B cells, the replication kinetics of rRSVs exhibited distinct characteristics. The extended virus, rRSV BA9 WT, reached its peak on day 4 (1.64 × 10^6^ cp/mL), and rRSV BA9 Δ7AA peaked on day 6 (2.70 × 10^5^ cp/mL), as illustrated in Figure 3b.

HRSV infection is known to stimulate the production of interferons (IFNs). HEp-2 and BEAS-2B cells were exposed to rRSVs at an MOI of 0.01, and supernatants were collected at 24 and 48 h post-infection. The ELISA results indicated that rRSV BA9 Δ7AA significantly increased the IFN-β levels in BEAS-2B cells at 48 h (154.33 pg/mL vs. 11.27 pg/mL) (Figure 3d). However, the secretion of IFN-α or IFN-λ was undetectable following rRSV infection. These findings suggest that compared to the non-extended virus, the G-extended virus markedly suppressed type I IFN expression in BEAS-2B cells.

To further investigate the distinct characteristics of the G gene extension in cellular responses, the cytopathic effect (CPE) induced by two recombinant viruses (rRSV BA9 WT and rRSV BA9 Δ7AA) was evaluated. As shown in Figure 3e,f, a distinct cytopathic effect was observed in HEp-2 cells, characterized by the fusion of adjacent cells and the subsequent formation of syncytia. Notably, the extension of seven amino acids into the G gene significantly influences the syncytia size, with markedly larger syncytia observed in cells infected with rRSV BA9 WT. Concentrated regions indicative of syncytia formation were also detected in BEAS-2B cells infected with rRSVs. In comparison to the non-extended virus, the G-extended virus predominantly induced the formation of larger syncytia. Beyond syncytia formation, rRSV infection in BEAS-2B cells elicited additional cytopathic effects, including cell rounding, and swelling and detachment of the monolayer from the cell culture flask surface. After six days post-infection, severe damage culminated in the complete loss of cell viability. These findings suggest that the extension of seven amino acids in the G gene facilitates the formation of larger syncytia.

### 2.3. Immunoplaque-Based Microneutralization Assay for the Identification of Antibodies

**Figure 4:**
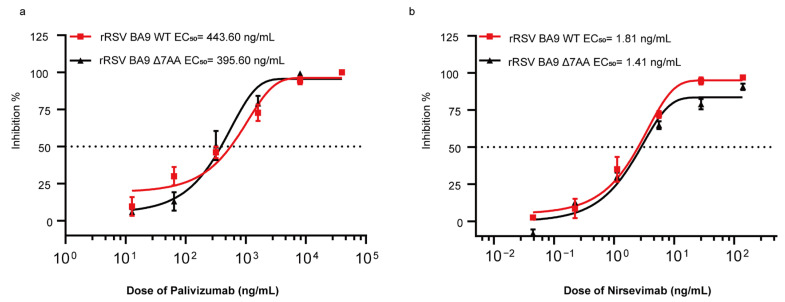
Dose–response curve of neutralizing activity of palivizumab or nirsevimab via microneutralization assay. Medium containing virus and 4-fold serial dilutions of palivizumab (**a**) and nirsevimab (**b**) was added to the cells. At 48 h post-infection, the half maximal effective concentration (EC_50_) was determined using a dose–response curve drawn based on viral titers (inhibition of infection) in infected cells using four-parameter nonlinear regression. Three independent experiments were performed; error bars represent SEM.

To assess the neutralization capacity of monoclonal antibodies against rRSVs (rRSV BA9 WT and rRSV BA9 Δ7AA) microneutralization assays were performed in HEp-2 cells. Two monoclonal antibodies, palivizumab and nirsevimab, were selected for the analysis. Viral replication inhibition was evaluated at 48 h post-infection by quantifying the reduction in viral plaque numbers relative to the control group treated with DMEM. As illustrated in Figure 4a,b, both monoclonal antibodies demonstrated neutralization activities in a dose-dependent manner. The half-maximal effective concentration (EC_50_) of palivizumab required to inhibit the replication of either rRSV BA9 WT or rRSV BA9 Δ7AA was determined to be 443.60 ng/mL and 395.60 ng/mL, respectively. For nirsevimab, the EC_50_ values for inhibiting the replication of rRSV BA9 WT and rRSV BA9 Δ7AA were 1.81 ng/mL and 1.41 ng/mL, respectively. These results suggest that the seven-amino-acid extension does not impact the virus’s neutralization susceptibility to either palivizumab or nirsevimab.

### 2.4. Phenotypic Characterization of Recombinant Viruses In Vivo

To assess the in vivo pathogenicity of rRSVs (rRSV BA9 WT and rRSV BA9 Δ7AA), eight-week-old BALB/c mice were intranasally infected with 10^6^ PFU/mouse. Body weight changes and lung viral loads were monitored over five consecutive days.

#### 2.4.1. Body Weight and Viral Loads

**Figure 5:**
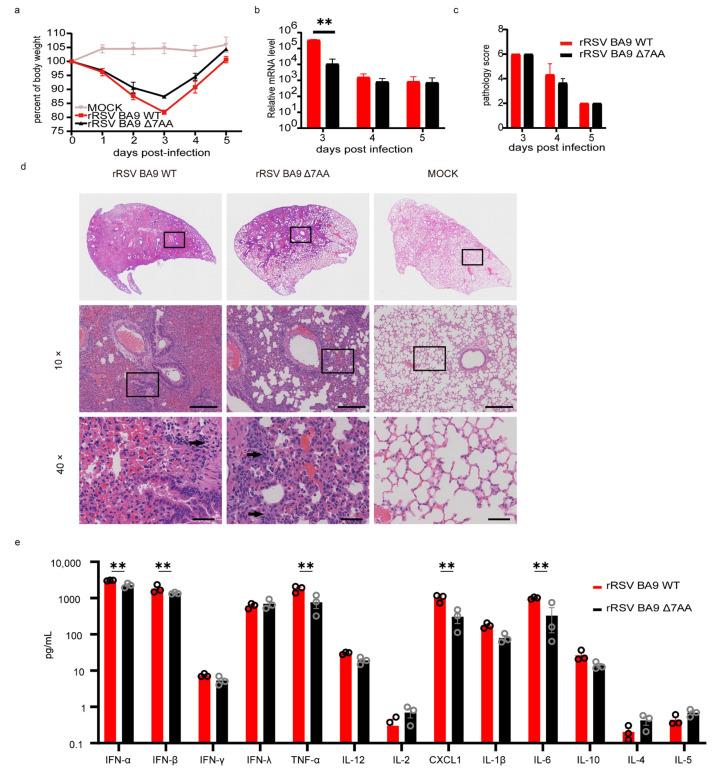
Biological characteristics of recombinant respiratory syncytial virus in vivo. Mice were either mock-(PBS) infected or infected with rRSVs (*n* = 3/group); (**a**) body weight was evaluated at the indicated days post-infection; (**b**) the presence of each virus load in lung of mice. **: *p*-value < 0.05; (**c**) the pathogenicity of mice lungs following RSV exposure; (**d**) histology of lung sections. Lungs were sectioned for hematoxylin and eosin (H&E) staining at 96 h; (**e**) cytokine concentrations in lung were measured by using ELISA at 72 h. The presence of cytokine in the BALF was determined using ELISA assay. **: *p*-value < 0.05.

All the infected mice exhibited significant weight loss starting on day 1 post-infection. Notably, mice infected with the extended virus (rRSV BA9 WT) displayed a greater reduction in body weight and slower recovery compared to those infected with rRSV BA9 Δ7AA on day 3 (Figure 5a).

Lung viral loads, measured via RT-qPCR, revealed significantly higher levels in the rRSV BA9 WT group compared to the other groups on day 3 post-infection (3.39 × 10^5^ vs. 1.08 × 10^4^, *p* < 0.05) (Figure 5b). However, no significant differences in viral loads were observed across the groups on days 4 and 5.

#### 2.4.2. Pulmonary Pathology

To evaluate the pathogenic effects on murine lungs following exposure to the HRSV, lung tissues collected on days 3, 4, and 5 post-infection were analyzed via H&E staining. On day 3, pronounced pathological changes were observed in all the infected groups. By day 4, the mice infected with rRSV BA9 WT exhibited the highest pathological damage scores, with lesions affecting over 50% of their lung tissue. These included significant inflammatory cell infiltration around blood vessels and alveoli (Figure 5c,d). These findings indicate that the G-extended virus induces more severe pulmonary pathology than a non-extended virus.

#### 2.4.3. Cytokine and Chemokine Profiles

To further investigate the inflammatory response induced by the recombinant viruses, we measured the levels of 13 cytokines and chemokines in the lungs of infected mice on day 3 post-infection, while the concentrations of cytokines and chemokines such as IFN-γ, IFN-λ, and IL-12 were comparable between the lungs of mice infected with the rRSV BA9 WT virus and those infected with the rRSV BA9 Δ7AA virus, the levels of IFN-α, IFN-β, TNF-α, CXCL-1, and IL-6 were significantly elevated in the lungs of mice infected with the rRSV BA9 WT virus compared to those infected with the rRSV BA9 Δ7AA virus (*p* < 0.05). Thus, the extended rRSV BA9 WT virus elicited a more pronounced inflammatory response in the lungs of infected mice than the rRSV BA9 Δ7AA virus, as illustrated in Figure 5e. These findings show that the G-extended virus causes more severe lung damage and adapts better to mice than the non-extended virus.

## 3. Discussion

The HRSV can cause severe lower respiratory tract infections, and a variety of factors may contribute to its clinical severity, including environmental, viral, and host factors [9]. Since its identification, the seven-amino-acid-extension variant has become widespread in China and around the world as an important evolutionary lineage of genotype BA9, and has shown a strong correlation with severe clinical manifestations [7]. This study compared the biological and immunological properties of the variants containing the seven-amino-acid extension and those without extension in the cellular and animal model, and confirmed that the extension of the G protein could enhance the virulence of the HRSV.

The present results show that the two rescued rRSVs replicated effectively in both HEp-2 and BEAS-2B cells. However, the two rescued rRSVs exhibited higher viral replication kinetics in HEp-2 cells compared to BEAS-2B cells, consistent with previous studies [10]. This difference may be attributed to the varying levels of interferon (IFN) secretion between these cell lines. BEAS-2B cells produced significantly higher IFN secretion than HEp-2, indicating a more robust antiviral activity. In particular, type I interferons (IFN-Is) play a critical role in the host defense by regulating the activity of the immune system against HRSV infection [11]. The G protein of the HRSV could influence the host IFN-I response by negatively regulating IFN-I signaling [12]. Therefore, different replication kinetics induced by the two rescued rRSVs were only observed in the BEAS-2B cells. The G-extended virus (with seven-amino-acid extension) replicates more permissively than non-extended viruses in BEAS-2B cells, with significantly lower IFN-β levels (11.27 pg/mL vs. 154.33 pg/mL). These findings suggest that the extended G protein may promote viral replication by inhibiting the innate immune response and suppressing IFN-β secretion.

The research on the interaction between the HRSV G protein and the host immune system has primarily focused on the conserved CX3C chemokine-like structure in the G protein. This CX3C motif enables the G protein to bind to the fractalkine receptor (CX3CR1) and modify the host’s immune response. For example, it suppresses type I and III IFN in airway epithelial cells following infection, impairing both innate and adaptive immunity [13]. In addition to the CX3C region, this study demonstrates that the extension of the G protein by seven amino acids at its C-terminus was also shown to inhibit IFN-I. This research enhances the understanding of G protein–host immune interactions, highlighting the potential role of these seven amino acids in modulating the immune response. Further studies are needed to determine whether these seven amino acids directly influence the virus–host interaction or indirectly affect immune responses through the CX3C region.

Generally, there is a correlation where larger plaques are associated with higher virulence [14,15,16,17]. In this study, we observed that the G-extended virus generated larger syncytia compared to the non-extended virus. This phenomenon is likely attributable to the modification of the G protein, which alters the relative expression levels of other proteins, such as the RSV fusion protein. The RSV fusion protein is known to facilitate the fusion of viral and host cell membranes and is directly associated with syncytium formation, thereby influencing the cytopathic effect [18]. Our findings also reveal that the G-extended virus exhibited higher expression levels of the fusion protein in HEp-2 cells compared to the non-extended virus, suggesting that the increased syncytium size in the G-extended virus may be due to elevated fusion protein expression (Figure 2c). These results align with earlier studies suggesting that codon positioning in the subtype A G gene might alter fusion protein expression levels [19].

Prior research has established a connection between O-glycosylation and viral maturation [20]. The glycosylation prediction results from this study suggest that the serine residues at positions 307 and 309 in the G protein of the extended virus are likely to be O-glycosylation sites. Nonetheless, our observations indicate that O-glycosylation does not influence viral maturation; rather, it solely modifies the molecular weight of the viral protein. Furthermore, in related members of the Pneumoviridae family, such as the Metapneumovirus, the G gene’s extension may add glycosylation sites, potentially altering the G protein’s extracellular structure and masking the fusion protein [21]. The neutralization assays conducted in this study did not demonstrate a significant shielding effect. Nonetheless, glycosylation may still affect other potential interactions involving viral proteins [19]. This study only predicts glycosylation sites based on sequences. Future investigations should employ cell lines deficient in O-glycosylation to clarify its functional implications.

The RSV primarily affects the respiratory system, with airway damage largely resulting from the host’s immune response rather than the virus itself, underscoring the pivotal role of the immune reaction in RSV pathogenesis [22]. This study also demonstrated that the seven-amino-acid extension modifies the virulence of the virus in mice, as indicated by the increased viral loads in the lungs and an augmented cytokine-mediated inflammatory response. Viral load is linked to the severity of human RSV disease, likely due to the interactions between viral factors and the host’s immune response [23,24]. The G-extended virus replicated more efficiently in the lungs of mice compared to the non-extended virus, eliciting a more pronounced inflammatory response. On day 3 post-infection, cytokine profiling revealed significantly higher levels of IFN-α, IFN-β, TNF-α, CXCL-1, and IL-6 in the lungs of mice infected with the G-extended virus compared to the Δ7AA virus. In contrast, rRSV BA9 Δ7AA induced higher IFN-β than the G-extended virus in the in vitro experiments, which may be because the in vitro experiments emphasized the innate immune response of the BEAS-2B cells, whereas the in vivo experiments were modulated by systemic factors such as the inflammatory microenvironment [25,26], temperature fluctuations [27], and multiple cell types in the lungs, including epithelial cells and macrophages [28,29], which collectively contribute to β-interferon secretion. Notably, the TNF-α and IL-6 expression levels were particularly elevated in mice infected with the extended virus. Similarly, elevated levels of TNF-α and IL-6 were observed in human patients with severe RSV infection [30,31,32]. High IL-6 expression levels have been detected in bronchoalveolar lavage fluid and nasal mucosa samples from patients with severe RSV infection [33,34]. Elevated levels of TNF-α have been documented in patients admitted to the ICU [35]. These increased TNF-α levels may exacerbate disease progression by enhancing cytokine synthesis through the NF-κB signaling pathway. The higher expression of cytokines associated with severe disease manifestations, along with the increased inflammatory cell infiltration and exacerbated pathological changes in the G-extended virus compared to the non-extended virus, may help explain the strong correlation between the extended variant strains and severe clinical outcomes in ICU patients.

## 4. Materials and Methods

### 4.1. Cell, Virus, and Plasmids

HEp-2 and BEAS-2B cell lines (ATCC, Rockefeller, MD, USA) were cultured in Dulbecco’s Modified Eagle Medium (DMEM) (Gibco BRL, Gaithersburg, MD, USA) supplemented with 10% fetal bovine serum (FBS, Gibco, Scoresby, Australia). BSR/T7-9 cells, generously provided by Professor W. Y. Zhu (CDC, Beijing, China), were also cultured in DMEM containing 10% FBS. The sequence of HRSV/SY/2021 was verified via Sanger sequencing (GenBank Accession No. PO765446). HEp-2 cells cultured in DMEM with 2% FBS and 0.2% sodium bicarbonate were used to propagate the virus. The full-length HRSV plasmid and helper plasmids (encoding the N, P, L, and M2-1 proteins, respectively) were synthesized by GenScript in Nanjing, China.

### 4.2. Recovery of Recombinant HRSV (rRSVs)

A full-length HRSV plasmid was constructed based on the sequence of the previously reported outbreak strain (GenBank Accession No. PO765446). The full-length HRSV plasmid was then transfected into BSR/T7-9 cells together with helper plasmids using Lipofectamine 3000 (Invitrogen, Carlsbad, CA, USA). Six hours after transfection, the supernatant was removed and then provided with DMEM containing 2% FBS. The viral supernatant was serially passaged on HEp-2 cells until the typical cytopathic effect (CPE) appeared, followed by subsequent experiments. Both rRSVs were confirmed to contain the correct sequences via next-generation sequencing (Supplementary Materials).

### 4.3. Western Blotting

HEp-2 cells were infected with the HRSV at a multiplicity of infection (MOI) of 0.01. After 48 h, proteins were extracted, and the solubilized proteins were collected from the supernatant following centrifugation at 12,000× *g* for 20 min. Proteins were then separated and transferred onto nitrocellulose membranes. A blocking solution consisting of 5% skimmed milk in PBS-Tween was employed. The antibodies used were palivizumab for the fusion protein, anti-RSV polyclonal antibody (Millipore; AB1128, Billerica, MA, USA) for the nucleocapsid protein, and HRP-conjugated RSV glycoprotein G antibody (Vazyme, Nanjing, China) along with HRP-conjugated beta-actin monoclonal antibody (BA3R). Western blots were visualized using ECL (Clarity Western ECL, Bio-Rad, Hercules, CA, USA), and chemiluminescence was detected using an imaging system.

### 4.4. Indirect Immunofluorescence Assay (IFA)

In a parallel experiment, HEp-2 or BEAS-2B cells were infected and fixed with 4% paraformaldehyde (PFA) for 15 min. Subsequently, the cells were washed and blocked with 5% bovine serum albumin at 37 °C for 1 h. After three additional phosphate-buffered saline (PBS) washes, the cells were incubated with a monoclonal antibody specific to the fusion protein at 37 °C for 1 h. After the initial procedures, the cells were incubated with FITC-conjugated Goat Anti-Human IgG (H + L) (Beyotime, Shanghai, China) at 37 °C for 45 min. The cell nuclei were stained with DAPI (Sigma, St. Louis, MO, USA), and fluorescent images were captured using a fluorescence microscope (Leica, Wetzlar, Germany).

### 4.5. Viral Multiple-Step Growth Curves Measured via RT-qPCR Assay

HEp-2 or BEAS-2B cells, cultured in 96-well plates, were infected with rRSVs at an MOI of 0.01. After a 1 h incubation period at 37 °C to facilitate viral adhesion, the cells were washed, and 100 μL of DMEM supplemented with 2% FBS was added to each well. Samples were collected at predetermined time points post-infection for real-time quantitative PCR (RT-qPCR) analysis. Subsequently, RT-PCR was conducted using the One Step PrimeScript™ RT-PCR kit (TaKaRa, Dalian, China) as previously described [3]. The viral multiple-step growth curve was illustrated using GraphPad Prism 8.0 software (GraphPad, La Jolla, CA, USA).

### 4.6. Plaque Assay

HEp-2 cells were seeded into 24-well plates with 3 × 10^5^ cells per well and incubated at 37 °C. Upon reaching approximately 90% confluency, the cells were washed with PBS and subsequently overlaid with 300 μL of virus-diluted supernatants. The cells were incubated at 37 °C for 60 min, after which the supernatants were removed. Subsequently, the cells were overlaid with 500 μL of microcrystalline cellulose-minimal essential medium and incubated at 37 °C for 120 h. The cells were then fixed and stained with 1% crystal violet. Plaques were enumerated, and viral titers were calculated in plaque-forming units per milliliter (PFU/mL).

### 4.7. Neutralization Experiments

A neutralization assay was performed using an immunoplaque-based microneutralization approach with the monoclonal antibodies palivizumab and nirsevimab. Antibodies were serially diluted and pre-incubated with the virus to allow neutralization prior to inoculation onto HEp-2 cells seeded in 96-well plates. This process was conducted in triplicate with incubation for one hour at 37 °C. Subsequently, DMEM containing 1.2% methylcellulose (Sigma) was added to the wells. Following a 2-day incubation, the cell monolayer was fixed with 4% PFA and incubated with palivizumab for 1 h. Subsequently, the cells were incubated with HRP-labeled goat anti-human IgG (Nakasugi Jinqiao, Beijing, China), enabling the assessment of the viral replication activity. The EC_50_ value was calculated by generating a dose–response curve for viral inhibition using four-parameter nonlinear regression.

### 4.8. Animal Experiments

Eight-week-old BALB/c mice were procured from the Charles River Experimental Animal Center in Beijing, China. All the procedures were approved by the Ethics Committee of the Chinese Center for Disease Control and Prevention (approval number 20220525058). The mice were intranasally infected with rRSV BA9 WT (1 × 10^6^ PFU) and rRSV BA9 Δ7AA (1 × 10^6^ PFU) for subsequent experimental analyses. Euthanasia was performed using carbon dioxide (CO_2_) asphyxiation, and mice were sacrificed at 3, 4, and 5 days post-infection for each designated time point.

### 4.9. Hematoxylin-Eosin Staining (H&E Staining)

Following euthanasia, lung tissues were excised. Lung tissue samples were fixed in 4% paraformaldehyde, followed by dehydration, clearing, paraffin embedding, and sectioning into 5 μm slices for histological examination. After dewaxing and rehydration, the tissue sections were stained using hematoxylin and eosin (H&E). The stained slides were examined under an optical microscope under consistent conditions. Briefly, the dewaxed and rehydrated sections were immersed in water, subjected to a graded ethanol dehydration series, rinsed with distilled water for 5 min, and stained with H&E staining kits for 3–5 min. Subsequent to this procedure, the samples were rinsed with water for 5 min, followed by differentiation in 1% ethanol-hydrochloric acid for 30 s. The samples were then washed with water for 30 s and further rinsed with distilled water for 5 s. Staining was performed using a 0.5% eosin solution. Dehydration was achieved through sequential immersion in ethanol. The samples were rendered transparent using xylene and sealed with neutral resin.

### 4.10. Pathology of Lung Injury Score

Lung injury was assessed based on the following pathological scoring criteria: 0 points indicated an intact alveolar wall without thickening, absence of inflammatory infiltrates, and no congestion; 1 point denoted slight diffuse inflammatory cell infiltration in the alveolar wall without thickening; 2 points represented marked and extensive inflammatory cell infiltration with slight thickening of the alveolar wall (1–2 times); 3 points indicated severe inflammatory cell infiltration with 2–3-fold thickening of the alveolar wall in specific areas; 4 points described severe inflammatory cell infiltration with marked thickening of the alveolar wall and 25–50% solidification of the lung tissue; and 5 points signified severe inflammatory cell infiltration with marked thickening of the alveolar wall and greater than 50% solidification of the lung tissue.

### 4.11. Enzyme-Linked Immunosorbent Assay (ELISA)

The expression levels of IFN-α, IFN-β, IFN-γ, IFN-λ, TNF-α, IL-12, IL-2, CXCL1, IL-1β, IL-6, IL-10, IL-4, and IL-5 in the samples were quantified using enzyme-linked immunosorbent assay (ELISA) kits procured from Solarbio, Beijing, China. All the procedures were conducted in accordance with the manufacturer’s instructions. Concentrations were ascertained by comparing the optical density readings to a standard curve.

### 4.12. Statistical Analysis

Statistical analyses were conducted using GraphPad Prism version 8.0. All the functional assays were conducted independently on a minimum of three occasions. The statistical significance for the comparisons between two groups was assessed using two-tailed Student’s *t*-tests. For multiple group comparisons, the statistical significance was evaluated using one-way analysis of variance (ANOVA). A *p*-value of less than 0.05 was regarded as indicative of statistical significance.

## 5. Conclusions

This study developed viral infectious clones from a severely ill preterm infant’s respiratory samples, with a primary focus on the C-terminal extension of the G protein of the RSV and its potential impact on viral virulence. Additionally, future studies should prioritize the global monitoring of these mutations to evaluate their potential contribution to the ongoing expansion of the epidemic. The evidence-based research connecting clinical cases and extended variant strains should be strengthened through the examination of disease pathological characteristics, inflammatory cell infiltration, and molecular markers. This comprehensive approach may yield valuable insights for the prevention and treatment of RSV-related diseases.

## Data Availability

The original contributions presented in this study are included in the article. Further inquiries can be directed to the corresponding authors.

## Supplementary Materials

The following supporting information can be downloaded at: https://www.mdpi.com/article/10.3390/ijms26052081/s1.
