# Supplementary material for: Enhanced Pathogenic Consequences Induced by a Seven-Amino-Acid Extension in the G Protein of the HRSV BA9 Genotype
Source: Int J Mol Sci. 2025 Feb 27;26(5):2081. doi: 10.3390/ijms26052081 (PMC11900327; doi:10.3390/ijms26052081)
Supplement: Supplementary file 1 [file ijms-26-02081-s001.zip › ijms-3459732-supplementary.pdf]

>Sequence-HRSV/SY/2021

TGCGAAAAAAAATGCGTACTACAAACTTGCACACTCGAAAAAAATGGGGCAAATAAGAATTTGATAAGTGCT
ATTTAAGTCTAACCTTTTTAATCAGAAATGGGGTGCAATTCACTGAGCATGATAAAGGTTAGATTACAAAATT
TGTTTGATAATGACGAAGTAGCATTGTTAAAAATAACATGTTATACTGACAAATTAATTCTTCTGACTAATGCAT
TAGCCAAAGCAACAATACATACAATTAAATTAAACGGCATAGTTTTTATACATGTTATAACAAGCAGTGAAGTG
TGCCCTGATAACAATATTGTAGTGAAATCTAACTTTACAACAATGCCAATATTACAAAATGGAGGATACATATG
GGAATTGATTGAATTGACACACTGCTCTCAATTAAATGGTCTAATAGATGATAATTGTGAAATCAAATTTTCTA
AAAGACTAAGTGACTCAGTAATGACTGATTATATGAATCAAATATCTGATTTACTTGGGCTTGATCTCCATTCAT
GAATTATGTTTAGTCTAATTCAATAGACATGTGTTTATTACCATTTTAGTTAATATAAAACCTCATCAAAGGGAA
ATGGGGCAAATAAACTCACCCAATCAATCAAACCATGAGCACTACAAACGACAACACCACCATGCAAAGATT
GATGATCACAGACATGAGACCCCTGTCGATGGATTCAATAATAACATCTCTCACTAAAGAAATCATCACACAC
AAATTCATATACTTGATAAACAATGAATGTATTGTAAGAAAACTCGATGAAAGACAAGCTACATTTACATTCCT
AGTCAATTATGAGATGAAGCTATTGCACAAAGTAGGGAGCACCAAATACAAGAAATACACCGAATATAATAC
AAAATATGGCACATTCCCTATGCCTATATTTATCAATCATGGCGGGTTTCTAGAATGTATTGGCATTAAGCCTAC
AAAACATACTCCTATAATATACAAATATGACCTCAACCCGTAACTTCCAACAAAAAAACCAACTCATCCAAACC
AAGCCATTCTCCAAACAACAATGCTCAACAGTTAAGAAGGAGCTAATCCATTTTAGTAATTAAAAATAAGGGT
GAAACCAGTAACATAAATTGGGGCAAATACAAAGATGGCTCTTAGCAAAGTCAAGTTGAATGATACATTAAA
TAAGGATCAGCTGCTGTCATCCAGCAAATACACTATTCAACGTAGTACAGGAGATAATATTGACACTCCCAATT
ATGATGTGCAAAAACACCTAAACAAACTATGTGGTATGCTATTAATCACTGAAGATGCAAATCATAAATTCACA
GGATTAATAGGTATGCTATATGCTATGTCCAGATTAGGAAGAGAAGACACTATAAAGATACTTAAAGATGCTG
GATATCATGTTAAAGCTAATGGAGTAGATATAACAACATATCGTCAAGATATAAATGGAAAGGAAATGAAATTC
GAAGTATTAACATTATCAAGCTTGACATCAGAAATACAAGTCAATATTGAGATAGAATCTAGAAAGTCCTACA
AAAAAATGCTAAAAGAGATGGGAGAAGTGGCTCCAGAATATAGGCATGATTCTCCAGACTGTGGGATGATA
ATACTGTGTATAGCTGCCCTTGTAATAACCAAATTAGCAGCAGGAGATAGATCAGGTCTTACAGCAGTAATTA
GGAGGGCAAACAATGTCTTAAAAAACGAAATAAAACGCTACAAGGGCCTAATACCAAAAGACATAGCCAAC
AGTTTTTATGAAGTGTTTGAAAAATACCCTCATCTTATAGATGTTTTTGTGCACTTTGGCATAGCACAATCATC
CACAAGAGGGGGCAGTAGAGTTGAAGGAATCTTTGCAGGATTGTTTATGAATGCCTATGGTTCAGGACAAG
TAATGCTAAGATGGGGAGTTTTAGCCAAATCTGTAAAAAATATCATGCTAGGACATGCTAGTGTCCAAGCAGA
AATGGAGCAAGTTGTGGAAGTCTATGAGTATGCACAGAAGTTGGGAGGAGAAGCTGGTTTCTACCATGTAT
TGAACAATCCAAAAGCATCATTGCTGTCATTAACTCAATTTCCTAACTTCTCAAGTGTGGTCCTAGGCAATGC
AGCAGGTCTAGGCATAATGGGAGAGTATAGAGGTACACCAAGAAACCAAGATCTCTATGATGCAGCCAAAG
CATATGCAGAGCAACTCAAAGAAAATGGAGTAATAAACTACAGTGTATTAGACTTAACAACAGAAGAATTGG
AAGCCATAAAGCATCAACTCAACCCCAAAGAAGATGACGTAGAGCTTTAAGTTAACAAAAAATACGGGGCA
AATAAGTCAACATGGAGAAGTTTGCACCTGAATTTCATGGAGAAGATGCAAATAACAAAGCTACCAAATTCC
TAGAATCAATAAAAGGCAAGTTTGCATCATCCAAAGATCCTAAGAAGAAAGATAGCATAATATCTGTCAACTC
AATAGACATAGAAGTCACTAAAGAGAGCCCGATAACATCTGGCACCAACATTATCAATCCAACAAGTGAAGC
CGACAGTACCCCAGAAACTAAAGCCAACTACCCAAGAAAACCCCTAGTAAGCTTCAAAGAAGATCTCACCC
CAAGTGATAACCCTTTCTCTAAGTTGTACAAAGAAACCATAGAAACATTTGATAACAATGAAGAAGAATCTAG
CTACTCATATGAGGAGATCAATGACCAAACAAATGACAACATTACAGCAAGACTAGATAGAATTGATGAAAA
ATTAAGTGAAATATTAGGAATGCTCCATACATTAGTAGTTGCAAGTGCAGGACCTACTTCGGCTCGTGACGGA
ATAAGAGATGCTATGGTTGGTCTAAGAGAAGAAATGATAGAAAAAATAAGAGCAGAAGCATTAATGACCAAT
GATAGGTTAGAGGCTATGGCAAGACTTAGGAATGAGGAAAGCGAAAAAATGGCAAAAGACACCTCAGATG

AAGTGTCTCTCAATCCAACCTCTAAAAAATTGAGTGACTTGTTGGAAGACAACGATAGCGACAATGATCTATC
ACTTGATGATTTTTGATCAGTGATCAACTCACTCAGCAATCAACAACATCAATAAAACAGACACCAATCCATT
GAATCAATTGCCAGACTGAAAAAACAAACATCCATCAGCAGAACCACCAACCAATCAATCAACCAATTGATC
AATCAGCACCCTGACAAAATTAACAATATAGTAACAAAAAAAGAACAAGATGGGGCAAATATGGAAACATAC
GTGAACAAGCTTCACGAAGGCTCCACATACACAGCAGCTGTCCAGTACAATGTTCTAGAAAAAGATGATGAT
CCCGCATCACTAACAATATGGGTGCCTATGTTCCAGTCATCTGTGCCAGCAGACTTGCTCATAAAAGAACTTG
CAAGCATCAACATACTAGTAAAGCAGATCTCTACGCCCAAAGGACCTTCACTACGAGTCACGATCAACTCAA
GAAGTGCTGTGCTGGCTCAAATGCCTAGTAATTTCACCATAAGTGCAAATGTATCATTAGATGAAAGAAGCAA
ATTAGCATATGATGTAACTACACCTTGTGAAATCAAAGCATGCAGTTTAACATGCTTAAAAGTAAAAAGTATGT
TAACTACAGTCAAAGATCTAACCATGAAGACATTCAACCCCACTCATGAGATCATTGCTCTATGTGAATTTGAA
AATATTATGACATCAAAAAGAGTAATAATACCAACCTATCTAAGATCAATCAGTGTCAAGAACAAAGATCTGA
ACTCACTGGAAAATATAGCAACCACCGAATTCAAAAATGCTATCACCAATGCTAAAATTATTCCCTATGCAGGA
TTAGTGTTAGTTATCACGGTTACTGATAATAAAGGAGCATTCAAGTATATCAAGCCACAGAGTCAATTTATAGT
GGATCTTGGTGCCTACCTAGAAAAAGAGAGCATATATTATGTGACTACTAATTGGAAGCATACAGCTACACGT
TTTTCAATCAAACCACTAGAGGATTAAACTCAATTATCAACATTGAATGACAGGTTCACATACATCCTCAACTG
CACACTGTATCTAAACATCATAAACATCTACACTACACACTTCATCACACAAACCAATCCCACTCAAAATCTAA
AATCACTTCCAGCCATTGTCTGCCAGACCTAGAGTGCGAATAGGTATATAAAACAAGAATATGGGGTAAATAG
ATATCAGTTAGAGTTCAACCAATCTCAACAACCATCTATACCGCCAATCCAATACATACATTGCAAATCTTAAAA
TGGGAAACACATCCATCACAATAGAATTCACAAGCAAATTTTGGCCTTATTTTACACTAATACACATGATCTTA
ACTCTAATCTCTTTACTAATTATAATCACTATTATGATTGCAATACTAAATAAGCTGAGTGAACATAAAATATTCT
GCAACAAAACTCTTGAGCAAGGACAGATGTATCAAATCGACACATAGTGTTCTCCCATTATGCTGTATCAAAT
CACAATCCTGTGTATATAAATAAACAAATCCAATCTTCTCACAGAGTCATGGCATCACAAAACCATGCCAACCA
TCATGGTAGCATAGAGTAGTTATTAAAAATTAACATAATGATGAATTATGAGTATGGGATCAAAAACAACATTG
GGGCAAATGCAACAATGTCCAAAAACAAGAATCAACGCACTGCCAGGACTCTAGAAAAGACCTGGGATACT
CTTAATCATCTAATTGTAATATCCTCTTGTTTATACAAATTAAATTTAAAATCTATAGCACAAATAGCACTATCAG
TTTTGGCAATGATAATCTCAACCTCTCTTATAATTGCAGCCATAATATTCATCATCTCTGCCAATCACAAAGTTA
CACTAACAACTGTCACAGTTCAAACAATAAAAAACCACACTGAGAAAAACATAACCACTTACCTTACTCAAG
TCTCGCCAGAAAGGGTTAGCCCATCCAAACAACCCACAGCCACACCGCCAATCCACACAAACTCAGCCACA
ATATCACCCAATACAAAATCAGATACACACCATACAACAACACAAACCAAAGGCACAATCTCTACTCCAACAC
AGAACAACAAGCCAAGCACAAAACCACGTCCAAAAAATCCACCAAAAAAAGATGATTACCATTTTGAAGTG
TTCAACTTTGTTCCCTGTAGTATATGTGGCAGCAATCAACTCTGCAAATCCATTTGCAAAACAATACCAAGCAA
TAAACCAAAGAAAAAACCAACCACAAAACCCACAAACAAACCACCTACCAAAACCACAAACAAAAGAGAC
CCCAAAACACTAGCCAAAACACCGAAAAAAGAAACCACCATTAACCCAACAAAAAAACCAACCCCCAAGAC
TACAGAAAGAGACACCAGCACACCACAATCCACTGTGCTCGACACAACCACATCAAAACACACAGAAAGG
GACACCAGCACCTCACAATCCATTGTGCTTGACACAACCGCATCAAAACACACAACCCAACAGCAATCTCTC
TACTCAACCATCCCTGAAAACACACCCAACTCCACACAAACACCCACAGCATCTGAGCCCTCTACATCAAATT
CTATCCAAAGACTCCAGTCATATGCTTAGTTATTTAAAAACTACATCTTAGCAAAGAACCGTGATCCCTTAAGC
AAGAACGAAATTAAATCTGGGGCAAATAACCATGGAGTTGCTGATCCATAGATCAAGTGCAATCTTCCTAACT
CTTGCTATTAATGCATTGTACCTTACCTCAAGTCAGAACATAACTGAGGAGTTTTACCAATCAACATGTAGTGC
AGTTAGCAGAGGTTACTTGAGTGCTTTAAGAACAGGTTGGTATACCAGTGTCATAACAATAGAATTAAGTAAT
ATAAAAGAAACCAAATGCAATGGAACTGACACTAAAGTTAAACTTATAAAACAAGAATTAGATAAGTATAAG
AATGCAGTAACTGAATTACAGTTACTTATGCAAAACACACCAGCTGTCAACAACCGGGCCAGAAGAGAAGC
ACCACAGTATATGAACTACACAATCAATACCACTAAAAACCTAAATGTATCAATAAGCAAGAAGAGGAAACG
AAGATTTCTGGGCTTCTTGTTAGGTGTAGGATCTGCAATAGCAAGTGGTATAGCTGTATCCAAAGTTCTACAC

CTTGAAGGAGAAGTGAACAAGATCAAAAATGCTTTGCAGCTTACAAACAAAGCTGTAGTCAGTCTATCAAAT
GGGGTCAGTGTTTTAACCAGCAGAGTGTTAGATCTCAAGAATTATATAAACAACCAATTATTACCTATGGTAA
ATCGACAGAGTTGTCGCATATCCAACATTGAGACAGTTATAGAATTCCAGCAGAAGAACAGCAGATTGTTGG
AAATCACCAGAGAATTTAGTGTCAATGCAGGTGTAACGACACCTTTAAGCACTTATATGTTAACAAACAGTGA
GTTACTATCATTAATCAATGATATGCCTATAACAAATGATCAGAAAAAATTAATGTCAAGCAATGTTCAGATAGT
AAGGCAACAAAGTTATTCTATCATGTCTATAATAAAGGAAGAAGTCCTTGCATATGTTGTACAGCTACCTATCT
ATGGTGTAATTGATACACCTTGCTGGAAATTACACACATCACCTCTGTGCACCACCAACATCAAAGAAGGATC
AAATATTTGTTTAACAAGGACTGATAGAGGATGGTATTGTGATAATGCAGGATCAGTATCCTTCTTTCCACAA
GCTGACACTTGTAAAGTACAGTCCAATCGAGTATTTTGTGACACTATGAACAGTTTGACATTACCAAGTGAAG
TCAGCCTTTGTAACACTGACATATTCAATTCCAAGTATGACTGCAAAATTATGACATCAAAAACAGACATAAG
CAGCTCAGTAATTACTTCTCTAGGAGCTATAGTGTCATGCTATGGCAAGACTAAATGTACTGCATCCAACAAA
AATCGTGGAATTATAAAGACATTTTCTAATGGTTGTGATTATGTGTCAAACAAAGGAGTAGATACTGTATCAGT
GGGCAACACTTTATATTATGTCAACAAGCTGGAAGGCAAAAACCTTTATGTAAAAGGGGAACCTATAATAAA
TTACTATGACCCTCTAGTGTTTCCTTCTGATGAGTTTGATGCATCAATATCTCAAGTCAATGAAAAAATTAATCA
AAGTTTAGCTTTTATTCATAGATCTGATGAATTATTACATAATGTAAATACTGGAAAATCTACTACAAATATTATG
ATAACTGCAATTATTATAGTAATCATTGTAGTATTGTTATCATTAATAGCTATTGGTTTACTGTTGTATTGCAAAG
CCAAAAACACACCAGTTACATTAAGCAAAGACCAACTAAGTGGAATCAATAATATTGCATTCAGCAAATAGAC
AAAAAACCACCTGATCATGTTCCAACAACAATCTGCTGACCATCAATCCCAAATCAACTTACAACAGATACTT
CAACATCACAGTACAGGCTGAATCATCTCCTCGCATCATGCTACCTACACAACTAAGCTAGATCCTTAATTCAT
AGTTACATAAAAGCCTCAAATATCGCAATCAACACTAAATCAACACATCATTTACAAAACTAACAGCTGGGGC
AAATATGTCGCGAAGAAATCCCTGCAAATTTGAGATTAGAGGTCATTGCTTGAATGGTAGAAGATGCCACTA
CAGTCATAATTACTTTGAATGGCCTCCTCATGCATTGCTAGTGAGGCAAAACTTCATGTTAAACAAGATACTCA
AGTCAATGGACAAGAGCATAGACACTTTGTCTGAAATAAGTGGAGCTGCTGAACTTGATAGAACAGAAGAA
TATGCTCTTGGTATAGTTGGAGTGCTAGAGAGTTACATAGGATCTATAAACAACATAACAAAACAATCAGCAT
GTGTTGCTATGAGTAAACTTCTTATTGAGATCAATAGTGATGACATTAAAAAGCTGAGAGATAATGAAGAACC
CAATTCACCTAAGATAAGAGTGTACAATACTGTTATATCATACATCGAGAGCAATAGAAAAAACAGCAAGCAA
ACCATCCATCTGCTTAAACGATTACCAGCAGACGTGCTGAAGAAGACAATAAAGAACACATTAGATATCCAC
AAAAGCATAACCATAAGCAACCCAAAAGAGTCAACCATAAGTGATCAAAATGACCAAACCAAAAATAATGAT
ATTACCGGATAAATATCCTTGTAGTATATCATCCATATTGATCTCAAGTGAAAGCATGATTGCTACATTCAACCAT
AAAGACATATTACAATTTAACCACAACCATTTAGATAACCACCAGTGTTTATTAAATCATATATTTGATGAAATT
CATTGGACACCTAAAAACTTATTAGATGCCACTCAACAATTTCTCCAACATCTTAACATCCCTGAAGATATATAT
ACAGTATATATATTAGTGTCATAATGCTTGATCATAACGATTCTATATCATCCAACCATAAAATTGTCTTAATAAA
GTCATGGGACAAAATGGATCCCATTATTAATGGAAGCTCTGCTAATGTATATCTAACTGATAGTTATCTAAAAG
GTGTTATCTCTTTTTCAGAATGTAATGCTTTAGGGAGTTACCTTTTTAACGGCCCTTATCTTAAAAATGATTATA
CCAACTTAATTAGTAGACAAAGCCCACTAATAGAGCATATGAATCTAAAAAAACTAACTATAACACAGTCATTA
ATATCTAGATACCATAAAGGTGAACTGAAATTAGAAGAACCAACTTATTTCCAGTCATTACTTATGACATATAA
AAGCATGTCCTCGTCTGAACAAATTGCTACAACTAACTTACTTAAAAAAATAATACGAAGAGCTATAGAAATA
AGTGATGTAAAGGTGTACGCCATCTTGAATAAACTAGGACTAAAGGAAAAGGACAGAGTTAAGCCCAACAA
CAATTCAGGTGATGAAAACTCAGTACTTACAACCATAATTAAAGATGATATACTCTCAGCTGTGGAAAACAAT
CAATCATATACAAATTCAGATAAAAATTACTCAGTAAATCAAAATATCAATATCAAAACAACACTCTTAAAAAA
GTTGATGTGTTCAATGCAACATCCTCCATCATGGTTAATACACTGGTTCAATTTATATACAAAATTAAATAACAT
ATTAACACAATATCGATCAAATGAGGTAAAAAGTCATGGGTTTATATTAATAGATAATCAAACTTTGAGTGGTT
TTCAGTTTATTTTAAATCAATATGGTTGCATTGTTTATCATAAAGGGCTCAAAAAAATTACAACTACTACATACA
ATCAATTTTTGACATGGAAAGACATCAGCCTCAGCAGATTAAATGTTTGCTTAATTACTTGGATAAGTAATTGT

TTAAATACATTAAATAAAAGCTTAGGGTTGAGATGCGGATTCAATAATGTTGTGCTATCACAATTATTCCTTTAC
GGAGATTGTATACTGAAATTATTTCATAATGAAGGCTTTTACATAATAAAAGAAGTAGAAGGATTTATTATGTC
TTTAATTCTAAACATAACAGAAGAAGATCAATTTAGGAAACGATTTTATAATAGCATGCTAAATAACATCACAG
ATGCAGCTATTAAGGCTCAAAAGGATCTACTATCAAGAGTATGTCACACTTTATTAGACAAGACAGTGTCTGA
TAATATCATAAATGGTAAATGGATAATTCTATTAAGTAAATTTCTTAAATTGATTAAGCTTGCAGGTGATAATAAT
CTCAATAACTTGAGTGAGCTATATTTTCTCTTCAGAATCTTTGGACATCCAATGGTTGATGAGAGACAAGCAA
TGGATGCTGTAAGAATTAACTGCAATGAAACTAAGTTCTATTTATTAAGTAGCCTAAGTACGTTGAGAGGTGC
TTTCATTTATAGAATCATAAAAGGGTTTGTAAATACCTACAACAGATGGCCCACTTTAAGGAATGCTATTGTCC
TACCTCTAAGATGGTTAAACTATTATAAACTTAATACTTATCCATCTCTACTTGAAATCACAGAAAATGATTTGA
TTATTTTATCAGGATTGCGGTTCTATCGTGAGTTTCATCTGCCTAAAAAAGTGGATCTTGAAATGATAATAAAT
GACAAAGCTATTTCTCCTCCAAAAGATCTAATATGGACTAGTTTTCCTAGAAATTACATGCCATCACATATACA
AAATTATATAGAACATGAAAAGTTGAAGTTCTCTGAAAGCGACAGATCAAGAAGAGTACTAGAGTATTACTT
GAGAGATAATAAATTCAATGAATGTGATCTATACAATTGTGTAGTTAATCAAAGCTATCTCAACAACTCTAATCA
TGTGGTATCACTAACTGGTAAAGAAAGAGAGCTCAGTGTGGGTAGAATGTTTGCTATGCAACCAGGTATGTT
TAGGCAAATCCAAATCTTAGCAGAAAAAATGATAGCCGAAAATATTTTACAATTCTTCCCTGAGAGTTTGACA
AGATATGGTGATCTAGAGCTTCAAAAGATATTAGAATTAAAAGCAGGAATAAGCAACAAGTCAAATCGTTATA
ATGATAACTACAACAATTATATCAGTAAATGTTCTATAATAACAGATCTTAGCAAATTTAATCAAGCATTTAGATA
TGAAACATCATGTGTCTGCAGTGATGTATTAGATGAACTGCATGGGGTACAATCTCTATTCTCTTGGTTGCATT
TAACAATACCTCTTGTCACAATAATATGTACATATAGACATGCACCTCCTTTTATAAAGGATCATGTTGTCAATCT
TAATGAAGTTGATGAACAAAGTGGGTTATACAGATATCATATGGGTGGTATTGAGGGCTGGTGTCAAAAACT
GTGGACCATTGAAGCCATATCATTATTAGATCTAATATCTCTTAAAGGTAAATTCTCCATCACAGCTCTGATAAA
TGGTGATAATCAGTCAATTGATATAAGTAAACCAGTTAGACTTATAGAGGGTCAGACCCATGCTCAAGCAGAT
TATTTGTTAGCATTAAATAGCCTTAAGTTGCTATATAAAGAGTATGCAGGCATAGGCCATAAGCTTAAGGGAA
CTGAGACCTATATATCTAGAGATATGCAGTTCATGAGCAAAACAATCCAGCACAATGGAGTGTACTATCCAGC
CAGTATCAAAAAAGTCCTGAGAGTAGGTCCATGGATAAATACAATACTTGATGATTTTAAAGTCAGTTTAGAA
TCTATAGGTAGCTTAACACAGGAGTTAGAATACAGAGGAGAAAGCTTATTATGCAGTTTAATATTTAGGAATA
TTTGGTTATACAATCAAATTGCTCTGCAACTCCGAAATCATGCTTTATGTAATAATAAGCTATATTTAGATATATT
GAAGGTATTAAAACACTTAAAAACCTTTTTTAATCTTGATAGTATCGATACGGCGTTATCATTGTATATGAACTT
GCCTATGCTGTTTGGTGGTGGTGATCCTAATTTGTTATATCGAAGCTTTTATAGGAGAACTCCAGACTTCCTTA
CAGAAGCTATAGTACATTCAGTGTTTGTGTTGAGCTATTATACTGGTCACGATCTACAAGATAAGCTCCAGGAT
CTTCCAGATGATAGACTGAACAAATTCTTGACTTGTGTCATCACATTTGATAAAAATCCAAATGCCGAGTTTGT
AACATTGATGAGGGATCCACAGGCTTTAGGGTCTGAAAGGCAAGCTAAAATTACTAGTGAGATTAATAGATT
AGCAGTAACAGAAGTCTTAAGTATAGCTCCAAACAAAATATTTTCTAAAAGTGCGCAACACTATACTACCACT
GAGATTGATCTAAATGACATTATGCAAAATATAGAACCAACTTACCCTCATGGATTAAGAGTTGTTTATGAAAG
TTTACCTTTTTATAAAGCAGAAAAAATAGTTAATCTTATATCAGGAACAAAATCCATAACTAATATACTTGAAAA
AACATCAGCGATAGATACAACTGATATTAATAGGGCTACTGATATGATGAGGAAAAATATAACCTTACTTATAA
GGATACTTCCACTAGATTGTAACAAAGACAAAAGAGAGTTATTAAGTTTAGAAAATCTTAGCATAACTGAATT
AAGCAAGTATGTAAGAGAAAGATCTTGGTCATTATCCAATATAGTAGGAGTAACATCACCAAGTATTATGTTCA
CAATGGACATTAAATATACAACTAGCACTATAGCCAGTGGTATAATTATAGAAAAATATAATGTTAATGGTTTAA
CTCGTGGTGAAAGAGGACCTACTAAACCATGGGTAGGTTCATCTACACAGGAGAAAAAAACAATGCCAGTG
TACAATAGACAAGTTTTAACCAAAAAGCAAAGAGACCAAATAGATTTATTAGCAAAATTAGACTGGGTATATG
CATCCATAGACAACAAAGATGAATTCATGGAAGAACTGAGTACTGGAACACTTGGACTGTCATATGAAAAGG
CCAAAAAGTTGTTTCCACAATATCTAAGTGTCAATTATTTACACCGGTTAACAGTCAGTAGTAGACCATGCGA
ATTCCCTGCCTCAATACCAGCTTATAGAACAACAAATTATCATTTTGATACTAGTCCTATCAATCATGTATTAACA

GAAAAGTATGGAGATGAAGATATCGACATTGTGTTTCAAAATTGCATAAGTTTTGGTCTTAGCTTGATGTCAG
TTGTGGAACAATTCACAAACATATGTCCTAATAGAATTATTCTCATACCGAAGCTGAATGAGATACATTTGATG
AAGCCTCCTATATTTACAGGAGATGTTGATATCATCAAGTTGAAGCAAGTGATACAAAAACAGCATATGTTCCT
ACCAGATAAAATAAGTTTAACCCAATATGTAGAATTATTCTTAAGTAACAAAGCACTTAAATCTGGATCTCACA
TCAACTCTAATTTAATATTAGCACATAAAATGTCTGATTATTTTCATAATGCGTATATTTTAAGTACTAATTTAGCT
GGTCATTGGATTCTGATTATTCAACTTATGAAAGATTCAAAAGGTATTTTTGAAAAAGATTGGGGAGAGGGG
TATATAACTGATCATATGTTCATTAATTTGAATGTTTTCTTTAATGCTTATAAGACTTATTTGCTATGTTTTCATAG
AGGTTATGGTAAAGCAAAATTAGAATGTGATATGAACACTTCAGATCTTCTTTGTGTTTTGGAGTTAATAGAC
AGTAGCTACTGGAAATCTATGTCTAAAGTTTTCCTAGAACAAAAAGTCATAAAATACATAGTCAATCAAGACA
CAAGTTTGCATAGAATAAAAGGTTGTCACAGTTTTAAGTTGTGGTTTTTAAAACGCCTTAATAATGCTAAATTT
ACCGTATGCCCTTGGGTTGTTAACATAGATTATCACCCAACACACATGAAAGCTATATTATCTTACATAGATTTA
GTTAGAATGGGGTTAATAAATGTAGATAAATTAACCATTAAAAATAAAAACAAATTCAATGATGAATTTTACAC
ATCAAATCTCTTTTACATTAGTTACAACTTTTCAGACAACACTCATCTGCTAACAAAACAAATAAGGATTGCTA
ATTCAGAATTAAAAGATAATTATAACAAACTATATAACCCAACCCCAGAAGCTTTAGAAAATGTATCATTAATCC
CTGTTAAAAGTAATAATAGAAACAAACCTAAATTTTGTATAAGTGGAAGTACTGAATCTATGATGACGTCAACA
TTCTCTAATAAAATGCATATTAAATCTTCCACTGTTACCACAAGATTCAATTATAGCAGACAAGACTTGTACAAT
TTATTTCCAATTGTTGTGATAGACAGGATTATAGATCATTCAGGTAATACAGAAAAATCTAACCAACTTTACAC
TACCACTTCACATCAGACATCTTTAGTAAGGAATAGTGCATCACTTTATTGCATGCTTCCTTGGCATCATGTCAA
TAGATTTAACTTTGTATTTAGTTCCACAGGATGCAAGATCAGTATAGAATATATTTTAAAAGATCTTAAGATTAA
AGATCCCAGTTGTATAGCATTCATAGGTGAAGGAGCTGGTAACTTATTATTACGTACGGTAGTAGAACTTCATC
CTGACATAAGATATATTTACAGAAGTTTAAAAGATTGCAATGATCATAGTTTACCTATTGAATTTCTAAGGTTAT
ACAACGGGCATATAAACATAGATTATGGTGAGAATTTAACCATTCCTGCTACAGATGCAACTAACAACATACAT
TGGTCTTATTTACACATAAAATTTGCAGAACCTATTAGTATCTTTGTCTGTGATGCTGAATTACCTGTCACAGCC
AATTGGAGTAAAATCATAATTGAATGGAGTAAGCATGTAAGAAAGTGCAAATACTGTTCTTCTGTAAATAGAT
GCATTTTAATTGCAAAATACCATGCTCAAGATGATATTGATTTCAAATTAGATAACATTACTATATTGAAAACTT
ATGTGTGCCTAGGTAGCAAGTTAAAAGGATCTGAAGTTTACTTAGTCCTTACAATAGGCCCTGCAAATATACT
TCCTGTTTTTGATGTTGTGCAAAATGCTAAATTGATTCTTTCAAGAACTAAAAATTTCATTATGCCTAAAAAGA
TTGACAAGGAATCTATCGATGCAAATATTAAAAGCTTAATACCTTTCCTTTGTTACCCTATAACAAAAAATGGA
ATTAAGACTTCATTGTCAAAATTGAAGAGTGTAGTTAATGGAGATATATTATCATATTCTATAGCTGGACGTAAT
GAAGTATTCAGCAACAAGCTTATAAACCACAAGCATATGAATATCTTAAAATGGCTGGATCATGTTTTAAACTT
TAGATCAGCTGAACTTAATTACAATCATTTATACATGATAGAGTCCACATATCCTTACTTGAGTGAATTATTAAAT
AGTTTAACAACCAATGAGCTCAAGAAGCTGATTAAAATAACAGGTAGTGTACTATACAACCTTCCTAATGAAC
AGTAACTTAAAATATCATTAACAAGTTTGGTCAAATTTAGATGCTAACACATTATTATATTATAGTTATTAAAAA
AATATGCAAACTTTTCAATAATTTAGCATATTGATTCCAAGATTATCATTTTAGTCTTAAGGGATTAAATAAAAG
TCTAAAACTAACAATCACACATGTGCATTTACAACAC

>Sequence-rRSV BA9 WT

TGCGAAAAAAAATGCGTACTACAAACTTGCACACTCGAAAAAAATGGGGCAAATAAGAATTTGATAAGTGCT
ATTTAAGTCTAACCTTTTTAATCAGAAATGGGGTGCAATTCACTGAGCATGATAAAGGTTAGATTACAAAATT
TGTTTGATAATGACGAAGTAGCATTGTTAAAAATAACATGTTATACTGACAAATTAATTCTTCTGACTAATGCAT
TAGCCAAAGCAACAATACATACAATTAAATTAAACGGCATAGTTTTTATACATGTTATAACAAGCAGTGAAGTG
TGCCCTGATAACAATATTGTAGTGAAATCTAACTTTACAACAATGCCAATATTACAAAATGGAGGATACATATG

GGAATTGATTGAATTGACACACTGCTCTCAATTAAATGGTCTAATAGATGATAATTGTGAAATCAAATTTTCTA
AAAGACTAAGTGACTCAGTAATGACTGATTATATGAATCAAATATCTGATTTACTTGGGCTTGATCTCCATTCAT
GAATTATGTTTAGTCTAATTCAATAGACATGTGTTTATTACCATTTTAGTTAATATAAAACCTCATCAAAGGGAA
ATGGGGCAAATAAACTCACCCAATCAATCAAACCATGAGCACTACAAACGACAACACCACCATGCAAAGATT
GATGATCACAGACATGAGACCCCTGTCGATGGATTCAATAATAACATCTCTCACTAAAGAAATCATCACACAC
AAATTCATATACTTGATAAACAATGAATGTATTGTAAGAAAACTCGATGAAAGACAAGCTACATTTACATTCCT
AGTCAATTATGAGATGAAGCTATTGCACAAAGTAGGGAGCACCAAATACAAGAAATACACCGAATATAATAC
AAAATATGGCACATTCCCTATGCCTATATTTATCAATCATGGCGGGTTTCTAGAATGTATTGGCATTAAGCCTAC
AAAACATACTCCTATAATATACAAATATGACCTCAACCCGTAACTTCCAACAAAAAAACCAACTCATCCAAACC
AAGCCATTCTCCAAACAACAATGCTCAACAGTTAAGAAGGAGCTAATCCATTTTAGTAATTAAAAATAAGGGT
GAAACCAGTAACATAAATTGGGGCAAATACAAAGATGGCTCTTAGCAAAGTCAAGTTGAATGATACATTAAA
TAAGGATCAGCTGCTGTCATCCAGCAAATACACTATTCAACGTAGTACAGGAGATAATATTGACACTCCCAATT
ATGATGTGCAAAAACACCTAAACAAACTATGTGGTATGCTATTAATCACTGAAGATGCAAATCATAAATTCACA
GGATTAATAGGTATGCTATATGCTATGTCCAGATTAGGAAGAGAAGACACTATAAAGATACTTAAAGATGCTG
GATATCATGTTAAAGCTAATGGAGTAGATATAACAACATATCGTCAAGATATAAATGGAAAGGAAATGAAATTC
GAAGTATTAACATTATCAAGCTTGACATCAGAAATACAAGTCAATATTGAGATAGAATCTAGAAAGTCCTACA
AAAAAATGCTAAAAGAGATGGGAGAAGTGGCTCCAGAATATAGGCATGATTCTCCAGACTGTGGGATGATA
ATACTGTGTATAGCTGCCCTTGTAATAACCAAATTAGCAGCAGGAGATAGATCAGGTCTTACAGCAGTAATTA
GGAGGGCAAACAATGTCTTAAAAAACGAAATAAAACGCTACAAGGGCCTAATACCAAAAGACATAGCCAAC
AGTTTTTATGAAGTGTTTGAAAAATACCCTCATCTTATAGATGTTTTTGTGCACTTTGGCATAGCACAATCATC
CACAAGAGGGGGCAGTAGAGTTGAAGGAATCTTTGCAGGATTGTTTATGAATGCCTATGGTTCAGGACAAG
TAATGCTAAGATGGGGAGTTTTAGCCAAATCTGTAAAAAATATCATGCTAGGACATGCTAGTGTCCAAGCAGA
AATGGAGCAAGTTGTGGAAGTCTATGAGTATGCACAGAAGTTGGGAGGAGAAGCTGGTTTCTACCATGTAT
TGAACAATCCAAAAGCATCATTGCTGTCATTAACTCAATTTCCTAACTTCTCAAGTGTGGTCCTAGGCAATGC
AGCAGGTCTAGGCATAATGGGAGAGTATAGAGGTACACCAAGAAACCAAGATCTCTATGATGCAGCCAAAG
CATATGCAGAGCAACTCAAAGAAAATGGAGTAATAAACTACAGTGTATTAGACTTAACAACAGAAGAATTGG
AAGCCATAAAGCATCAACTCAACCCCAAAGAAGATGACGTAGAGCTTTAAGTTAACAAAAAATACGGGGCA
AATAAGTCAACATGGAGAAGTTTGCACCTGAATTTCATGGAGAAGATGCAAATAACAAAGCTACCAAATTCC
TAGAATCAATAAAAGGCAAGTTTGCATCATCCAAAGATCCTAAGAAGAAAGATAGCATAATATCTGTCAACTC
AATAGACATAGAAGTCACTAAAGAGAGCCCGATAACATCTGGCACCAACATTATCAATCCAACAAGTGAAGC
CGACAGTACCCCAGAAACTAAAGCCAACTACCCAAGAAAACCCCTAGTAAGCTTCAAAGAAGATCTCACCC
CAAGTGATAACCCTTTCTCTAAGTTGTACAAAGAAACCATAGAAACATTTGATAACAATGAAGAAGAATCTAG
CTACTCATATGAGGAGATCAATGACCAAACAAATGACAACATTACAGCAAGACTAGATAGAATTGATGAAAA
ATTAAGTGAAATATTAGGAATGCTCCATACATTAGTAGTTGCAAGTGCAGGACCTACTTCGGCTCGTGACGGA
ATAAGAGATGCTATGGTTGGTCTAAGAGAAGAAATGATAGAAAAAATAAGAGCAGAAGCATTAATGACCAAT
GATAGGTTAGAGGCTATGGCAAGACTTAGGAATGAGGAAAGCGAAAAAATGGCAAAAGACACCTCAGATG
AAGTGTCTCTCAATCCAACCTCTAAAAAATTGAGTGACTTGTTGGAAGACAACGATAGCGACAATGATCTATC
ACTTGATGATTTTTGATCAGTGATCAACTCACTCAGCAATCAACAACATCAATAAAACAGACACCAATCCATT
GAATCAATTGCCAGACTGAAAAAACAAACATCCATCAGCAGAACCACCAACCAATCAATCAACCAATTGATC
AATCAGCACCCTGACAAAATTAACAATATAGTAACAAAAAAAGAACAAGATGGGGCAAATATGGAAACATAC
GTGAACAAGCTTCACGAAGGCTCCACATACACAGCAGCTGTCCAGTACAATGTTCTAGAAAAAGATGATGAT
CCCGCATCACTAACAATATGGGTGCCTATGTTCCAGTCATCTGTGCCAGCAGACTTGCTCATAAAAGAACTTG
CAAGCATCAACATACTAGTAAAGCAGATCTCTACGCCCAAAGGACCTTCACTACGAGTCACGATCAACTCAA
GAAGTGCTGTGCTGGCTCAAATGCCTAGTAATTTCACCATAAGTGCAAATGTATCATTAGATGAAAGAAGCAA

ATTAGCATATGATGTAACTACACCTTGTGAAATCAAAGCATGCAGTTTAACATGCTTAAAAGTAAAAAGTATGT
TAACTACAGTCAAAGATCTAACCATGAAGACATTCAACCCCACTCATGAGATCATTGCTCTATGTGAATTTGAA
AATATTATGACATCAAAAAGAGTAATAATACCAACCTATCTAAGATCAATCAGTGTCAAGAACAAAGATCTGA
ACTCACTGGAAAATATAGCAACCACCGAATTCAAAAATGCTATCACCAATGCTAAAATTATTCCCTATGCAGGA
TTAGTGTTAGTTATCACGGTTACTGATAATAAAGGAGCATTCAAGTATATCAAGCCACAGAGTCAATTTATAGT
GGATCTTGGTGCCTACCTAGAAAAAGAGAGCATATATTATGTGACTACTAATTGGAAGCATACAGCTACACGT
TTTTCAATCAAACCACTAGAGGATTAAACTCAATTATCAACATTGAATGACAGGTTCACATACATCCTCAACTG
CACACTGTATCTAAACATCATAAACATCTACACTACACACTTCATCACACAAACCAATCCCACTCAAAATCTAA
AATCACTTCCAGCCATTGTCTGCCAGACCTAGAGTGCGAATAGGTATATAAAACAAGAATATGGGGTAAATAG
ATATCAGTTAGAGTTCAACCAATCTCAACAACCATCTATACCGCCAATCCAATACATACATTGCAAATCTTAAAA
TGGGAAACACATCCATCACAATAGAATTCACAAGCAAATTTTGGCCTTATTTTACACTAATACACATGATCTTA
ACTCTAATCTCTTTACTAATTATAATCACTATTATGATTGCAATACTAAATAAGCTGAGTGAACATAAAATATTCT
GCAACAAAACTCTTGAGCAAGGACAGATGTATCAAATCGACACATAGTGTTCTCCCATTATGCTGTATCAAAT
CACAATCCTGTGTATATAAATAAACAAATCCAATCTTCTCACAGAGTCATGGCATCACAAAACCATGCCAACCA
TCATGGTAGCATAGAGTAGTTATTAAAAATTAACATAATGATGAATTATGAGTATGGGATCAAAAACAACATTG
GGGCAAATGCAACAATGTCCAAAAACAAGAATCAACGCACTGCCAGGACTCTAGAAAAGACCTGGGATACT
CTTAATCATCTAATTGTAATATCCTCTTGTTTATACAAATTAAATTTAAAATCTATAGCACAAATAGCACTATCAG
TTTTGGCAATGATAATCTCAACCTCTCTTATAATTGCAGCCATAATATTCATCATCTCTGCCAATCACAAAGTTA
CACTAACAACTGTCACAGTTCAAACAATAAAAAACCACACTGAGAAAAACATAACCACTTACCTTACTCAAG
TCTCGCCAGAAAGGGTTAGCCCATCCAAACAACCCACAGCCACACCGCCAATCCACACAAACTCAGCCACA
ATATCACCCAATACAAAATCAGATACACACCATACAACAACACAAACCAAAGGCACAATCTCTACTCCAACAC
AGAACAACAAGCCAAGCACAAAACCACGTCCAAAAAATCCACCAAAAAAAGATGATTACCATTTTGAAGTG
TTCAACTTTGTTCCCTGTAGTATATGTGGCAGCAATCAACTCTGCAAATCCATTTGCAAAACAATACCAAGCAA
TAAACCAAAGAAAAAACCAACCACAAAACCCACAAACAAACCACCTACCAAAACCACAAACAAAAGAGAC
CCCAAAACACTAGCCAAAACACCGAAAAAAGAAACCACCATTAACCCAACAAAAAAACCAACCCCCAAGAC
TACAGAAAGAGACACCAGCACACCACAATCCACTGTGCTCGACACAACCACATCAAAACACACAGAAAGG
GACACCAGCACCTCACAATCCATTGTGCTTGACACAACCGCATCAAAACACACAACCCAACAGCAATCTCTC
TACTCAACCATCCCTGAAAACACACCCAACTCCACACAAACACCCACAGCATCTGAGCCCTCTACATCAAATT
CTATCCAAAGACTCCAGTCATATGCTTAGTTATTTAAAAACTACATCTTAGCAAAGAACCGTGATCCCTTAAGC
AAGAACGAAATTAAATCTGGGGCAAATAACCATGGAGTTGCTGATCCATAGATCAAGTGCAATCTTCCTAACT
CTTGCTATTAATGCATTGTACCTTACCTCAAGTCAGAACATAACTGAGGAGTTTTACCAATCAACATGTAGTGC
AGTTAGCAGAGGTTACTTGAGTGCTTTAAGAACAGGTTGGTATACCAGTGTCATAACAATAGAATTAAGTAAT
ATAAAAGAAACCAAATGCAATGGAACTGACACTAAAGTTAAACTTATAAAACAAGAATTAGATAAGTATAAG
AATGCAGTAACTGAATTACAGTTACTTATGCAAAACACACCAGCTGTCAACAACCGGGCCAGAAGAGAAGC
ACCACAGTATATGAACTACACAATCAATACCACTAAAAACCTAAATGTATCAATAAGCAAGAAGAGGAAACG
AAGATTTCTGGGCTTCTTGTTAGGTGTAGGATCTGCAATAGCAAGTGGTATAGCTGTATCCAAAGTTCTACAC
CTTGAAGGAGAAGTGAACAAGATCAAAAATGCTTTGCAGCTTACAAACAAAGCTGTAGTCAGTCTATCAAAT
GGGGTCAGTGTTTTAACCAGCAGAGTGTTAGATCTCAAGAATTATATAAACAACCAATTATTACCTATGGTAA
ATCGACAGAGTTGTCGCATATCCAACATTGAGACAGTTATAGAATTCCAGCAGAAGAACAGCAGATTGTTGG
AAATCACCAGAGAATTTAGTGTCAATGCAGGTGTAACGACACCTTTAAGCACTTATATGTTAACAAACAGTGA
GTTACTATCATTAATCAATGATATGCCTATAACAAATGATCAGAAAAAATTAATGTCAAGCAATGTTCAGATAGT
AAGGCAACAAAGTTATTCTATCATGTCTATAATAAAGGAAGAAGTCCTTGCATATGTTGTACAGCTACCTATCT
ATGGTGTAATTGATACACCTTGCTGGAAATTACACACATCACCTCTGTGCACCACCAACATCAAAGAAGGATC
AAATATTTGTTTAACAAGGACTGATAGAGGATGGTATTGTGATAATGCAGGATCAGTATCCTTCTTTCCACAA

GCTGACACTTGTAAAGTACAGTCCAATCGAGTATTTTGTGACACTATGAACAGTTTGACATTACCAAGTGAAG
TCAGCCTTTGTAACACTGACATATTCAATTCCAAGTATGACTGCAAAATTATGACATCAAAAACAGACATAAG
CAGCTCAGTAATTACTTCTCTAGGAGCTATAGTGTCATGCTATGGCAAGACTAAATGTACTGCATCCAACAAA
AATCGTGGAATTATAAAGACATTTTCTAATGGTTGTGATTATGTGTCAAACAAAGGAGTAGATACTGTATCAGT
GGGCAACACTTTATATTATGTCAACAAGCTGGAAGGCAAAAACCTTTATGTAAAAGGGGAACCTATAATAAA
TTACTATGACCCTCTAGTGTTTCCTTCTGATGAGTTTGATGCATCAATATCTCAAGTCAATGAAAAAATTAATCA
AAGTTTAGCTTTTATTCATAGATCTGATGAATTATTACATAATGTAAATACTGGAAAATCTACTACAAATATTATG
ATAACTGCAATTATTATAGTAATCATTGTAGTATTGTTATCATTAATAGCTATTGGTTTACTGTTGTATTGCAAAG
CCAAAAACACACCAGTTACATTAAGCAAAGACCAACTAAGTGGAATCAATAATATTGCATTCAGCAAATAGAC
AAAAAACCACCTGATCATGTTCCAACAACAATCTGCTGACCATCAATCCCAAATCAACTTACAACAGATACTT
CAACATCACAGTACAGGCTGAATCATCTCCTCGCATCATGCTACCTACACAACTAAGCTAGATCCTTAATTCAT
AGTTACATAAAAGCCTCAAATATCGCAATCAACACTAAATCAACACATCATTTACAAAACTAACAGCTGGGGC
AAATATGTCGCGAAGAAATCCCTGCAAATTTGAGATTAGAGGTCATTGCTTGAATGGTAGAAGATGCCACTA
CAGTCATAATTACTTTGAATGGCCTCCTCATGCATTGCTAGTGAGGCAAAACTTCATGTTAAACAAGATACTCA
AGTCAATGGACAAGAGCATAGACACTTTGTCTGAAATAAGTGGAGCTGCTGAACTTGATAGAACAGAAGAA
TATGCTCTTGGTATAGTTGGAGTGCTAGAGAGTTACATAGGATCTATAAACAACATAACAAAACAATCAGCAT
GTGTTGCTATGAGTAAACTTCTTATTGAGATCAATAGTGATGACATTAAAAAGCTGAGAGATAATGAAGAACC
CAATTCACCTAAGATAAGAGTGTACAATACTGTTATATCATACATCGAGAGCAATAGAAAAAACAGCAAGCAA
ACCATCCATCTGCTTAAACGATTACCAGCAGACGTGCTGAAGAAGACAATAAAGAACACATTAGATATCCAC
AAAAGCATAACCATAAGCAACCCAAAAGAGTCAACCATAAGTGATCAAAATGACCAAACCAAAAATAATGAT
ATTACCGGATAAATATCCTTGTAGTATATCATCCATATTGATCTCAAGTGAAAGCATGATTGCTACATTCAACCAT
AAAGACATATTACAATTTAACCACAACCATTTAGATAACCACCAGTGTTTATTAAATCATATATTTGATGAAATT
CATTGGACACCTAAAAACTTATTAGATGCCACTCAACAATTTCTCCAACATCTTAACATCCCTGAAGATATATAT
ACAGTATATATATTAGTGTCATAATGCTTGATCATAACGATTCTATATCATCCAACCATAAAATTGTCTTAATAAA
GTCATGGGACAAAATGGATCCCATTATTAATGGTAGCTCTGCTAATGTATATCTAACTGATAGTTATCTAAAAG
GTGTTATCTCTTTTTCAGAATGTAATGCTTTAGGGAGTTACCTTTTTAACGGCCCTTATCTTAAAAATGATTATA
CCAACTTAATTAGTAGACAAAGCCCACTAATAGAGCATATGAATCTAAAAAAACTAACTATAACACAGTCATTA
ATATCTAGATACCATAAAGGTGAACTGAAATTAGAAGAACCAACTTATTTCCAGTCATTACTTATGACATATAA
AAGCATGTCCTCGTCTGAACAAATTGCTACAACTAACTTACTTAAAAAAATAATACGAAGAGCTATAGAAATA
AGTGATGTAAAGGTGTACGCCATCTTGAATAAACTAGGACTAAAGGAAAAGGACAGAGTTAAGCCCAACAA
CAATTCAGGTGATGAAAACTCAGTACTTACAACCATAATTAAAGATGATATACTCTCAGCTGTGGAAAACAAT
CAATCATATACAAATTCAGATAAAAATTACTCAGTAAATCAAAATATCAATATCAAAACAACACTCTTAAAAAA
GTTGATGTGTTCAATGCAACATCCTCCATCATGGTTAATACACTGGTTCAATTTATATACAAAATTAAATAACAT
ATTAACACAATATCGATCAAATGAGGTAAAAAGTCATGGGTTTATATTAATAGATAATCAAACTTTGAGTGGTT
TTCAGTTTATTTTAAATCAATATGGTTGCATTGTTTATCATAAAGGGCTCAAAAAAATTACAACTACTACATACA
ATCAATTTTTGACATGGAAAGACATCAGCCTCAGCAGATTAAATGTTTGCTTAATTACTTGGATAAGTAATTGT
TTAAATACATTAAATAAAAGCTTAGGGTTGAGATGCGGATTCAATAATGTTGTGCTATCACAATTATTCCTTTAC
GGAGATTGTATACTGAAATTATTTCATAATGAAGGCTTTTACATAATAAAAGAAGTAGAAGGATTTATTATGTC
TTTAATTCTAAACATAACAGAAGAAGATCAATTTAGGAAACGATTTTATAATAGCATGCTAAATAACATCACAG
ATGCAGCTATTAAGGCTCAAAAGGATCTACTATCAAGAGTATGTCACACTTTATTAGACAAGACAGTGTCTGA
TAATATCATAAATGGTAAATGGATAATTCTATTAAGTAAATTTCTTAAATTGATTAAGCTTGCAGGTGATAATAAT
CTCAATAACTTGAGTGAGCTATATTTTCTCTTCAGAATCTTTGGACATCCAATGGTTGATGAGAGACAAGCAA
TGGATGCTGTAAGAATTAACTGCAATGAAACTAAGTTCTATTTATTAAGTAGCCTAAGTACGTTGAGAGGTGC
TTTCATTTATAGAATCATAAAAGGGTTTGTAAATACCTACAACAGATGGCCCACTTTAAGGAATGCTATTGTCC

TACCTCTAAGATGGTTAAACTATTATAAACTTAATACTTATCCATCTCTACTTGAAATCACAGAAAATGATTTGA
TTATTTTATCAGGATTGCGGTTCTATCGTGAGTTTCATCTGCCTAAAAAAGTGGATCTTGAAATGATAATAAAT
GACAAAGCTATTTCTCCTCCAAAAGATCTAATATGGACTAGTTTTCCTAGAAATTACATGCCATCACATATACA
AAATTATATAGAACATGAAAAGTTGAAGTTCTCTGAAAGCGACAGATCAAGAAGAGTACTAGAGTATTACTT
GAGAGATAATAAATTCAATGAATGTGATCTATACAATTGTGTAGTTAATCAAAGCTATCTCAACAACTCTAATCA
TGTGGTATCACTAACTGGTAAAGAAAGAGAGCTCAGTGTGGGTAGAATGTTTGCTATGCAACCAGGTATGTT
TAGGCAAATCCAAATCTTAGCAGAAAAAATGATAGCCGAAAATATTTTACAATTCTTCCCTGAGAGTTTGACA
AGATATGGTGATCTAGAGCTTCAAAAGATATTAGAATTAAAAGCAGGAATAAGCAACAAGTCAAATCGTTATA
ATGATAACTACAACAATTATATCAGTAAATGTTCTATAATAACAGATCTTAGCAAATTTAATCAAGCATTTAGATA
TGAAACATCATGTGTCTGCAGTGATGTATTAGATGAACTGCATGGGGTACAATCTCTATTCTCTTGGTTGCATT
TAACAATACCTCTTGTCACAATAATATGTACATATAGACATGCACCTCCTTTTATAAAGGATCATGTTGTCAATCT
TAATGAAGTTGATGAACAAAGTGGGTTATACAGATATCATATGGGTGGTATTGAGGGCTGGTGTCAAAAACT
GTGGACCATTGAAGCCATATCATTATTAGATCTAATATCTCTTAAAGGTAAATTCTCCATCACAGCTCTGATAAA
TGGTGATAATCAGTCAATTGATATAAGTAAACCAGTTAGACTTATAGAGGGTCAGACCCATGCTCAAGCAGAT
TATTTGTTAGCATTAAATAGCCTTAAGTTGCTATATAAAGAGTATGCAGGCATAGGCCATAAGCTTAAGGGAA
CTGAGACCTATATATCTAGAGATATGCAGTTCATGAGCAAAACAATCCAGCACAATGGAGTGTACTATCCAGC
CAGTATCAAAAAAGTCCTGAGAGTAGGTCCATGGATAAATACAATACTTGATGATTTTAAAGTCAGTTTAGAA
TCTATAGGTAGCTTAACACAGGAGTTAGAATACAGAGGAGAAAGCTTATTATGCAGTTTAATATTTAGGAATA
TTTGGTTATACAATCAAATTGCTCTGCAACTCCGAAATCATGCTTTATGTAATAATAAGCTATATTTAGATATATT
GAAGGTATTAAAACACTTAAAAACCTTTTTTAATCTTGATAGTATCGATACGGCGTTATCATTGTATATGAACTT
GCCTATGCTGTTTGGTGGTGGTGATCCTAATTTGTTATATCGAAGCTTTTATAGGAGAACTCCAGACTTCCTTA
CAGAAGCTATAGTACATTCAGTGTTTGTGTTGAGCTATTATACTGGTCACGATCTACAAGATAAGCTCCAGGAT
CTTCCAGATGATAGACTGAACAAATTCTTGACTTGTGTCATCACATTTGATAAAAATCCAAATGCCGAGTTTGT
AACATTGATGAGGGATCCACAGGCTTTAGGGTCTGAAAGGCAAGCTAAAATTACTAGTGAGATTAATAGATT
AGCAGTAACAGAAGTCTTAAGTATAGCTCCAAACAAAATATTTTCTAAAAGTGCGCAACACTATACTACCACT
GAGATTGATCTAAATGACATTATGCAAAATATAGAACCAACTTACCCTCATGGATTAAGAGTTGTTTATGAAAG
TTTACCTTTTTATAAAGCAGAAAAAATAGTTAATCTTATATCAGGAACAAAATCCATAACTAATATACTTGAAAA
AACATCAGCGATAGATACAACTGATATTAATAGGGCTACTGATATGATGAGGAAAAATATAACCTTACTTATAA
GGATACTTCCACTAGATTGTAACAAAGACAAAAGAGAGTTATTAAGTTTAGAAAATCTTAGCATAACTGAATT
AAGCAAGTATGTAAGAGAAAGATCTTGGTCATTATCCAATATAGTAGGAGTAACATCACCAAGTATTATGTTCA
CAATGGACATTAAATATACAACTAGCACTATAGCCAGTGGTATAATTATAGAAAAATATAATGTTAATGGTTTAA
CTCGTGGTGAAAGAGGACCTACTAAACCATGGGTAGGTTCATCTACACAGGAGAAAAAAACAATGCCAGTG
TACAATAGACAAGTTTTAACCAAAAAGCAAAGAGACCAAATAGATTTATTAGCAAAATTAGACTGGGTATATG
CATCCATAGACAACAAAGATGAATTCATGGAAGAACTGAGTACTGGAACACTTGGACTGTCATATGAAAAGG
CCAAAAAGTTGTTTCCACAATATCTAAGTGTCAATTATTTACACCGGTTAACAGTCAGTAGTAGACCATGCGA
ATTCCCTGCCTCAATACCAGCTTATAGAACAACAAATTATCATTTTGATACTAGTCCTATCAATCATGTATTAACA
GAAAAGTATGGAGATGAAGATATCGACATTGTGTTTCAAAATTGCATAAGTTTTGGTCTTAGCTTGATGTCAG
TTGTGGAACAATTCACAAACATATGTCCTAATAGAATTATTCTCATACCGAAGCTGAATGAGATACATTTGATG
AAGCCTCCTATATTTACAGGAGATGTTGATATCATCAAGTTGAAGCAAGTGATACAAAAACAGCATATGTTCCT
ACCAGATAAAATAAGTTTAACCCAATATGTAGAATTATTCTTAAGTAACAAAGCACTTAAATCTGGATCTCACA
TCAACTCTAATTTAATATTAGCACATAAAATGTCTGATTATTTTCATAATGCGTATATTTTAAGTACTAATTTAGCT
GGTCATTGGATTCTGATTATTCAACTTATGAAAGATTCAAAAGGTATTTTTGAAAAAGATTGGGGAGAGGGG
TATATAACTGATCATATGTTCATTAATTTGAATGTTTTCTTTAATGCTTATAAGACTTATTTGCTATGTTTTCATAG
AGGTTATGGTAAAGCAAAATTAGAATGTGATATGAACACTTCAGATCTTCTTTGTGTTTTGGAGTTAATAGAC

AGTAGCTACTGGAAATCTATGTCTAAAGTTTTCCTAGAACAAAAAGTCATAAAATACATAGTCAATCAAGACA
CAAGTTTGCATAGAATAAAAGGTTGTCACAGTTTTAAGTTGTGGTTTTTAAAACGCCTTAATAATGCTAAATTT
ACCGTATGCCCTTGGGTTGTTAACATAGATTATCACCCAACACACATGAAAGCTATATTATCTTACATAGATTTA
GTTAGAATGGGGTTAATAAATGTAGATAAATTAACCATTAAAAATAAAAACAAATTCAATGATGAATTTTACAC
ATCAAATCTCTTTTACATTAGTTACAACTTTTCAGACAACACTCATCTGCTAACAAAACAAATAAGGATTGCTA
ATTCAGAATTAAAAGATAATTATAACAAACTATATAACCCAACCCCAGAAGCTTTAGAAAATGTATCATTAATCC
CTGTTAAAAGTAATAATAGAAACAAACCTAAATTTTGTATAAGTGGAAGTACTGAATCTATGATGACGTCAACA
TTCTCTAATAAAATGCATATTAAATCTTCCACTGTTACCACAAGATTCAATTATAGCAGACAAGACTTGTACAAT
TTATTTCCAATTGTTGTGATAGACAGGATTATAGATCATTCAGGTAATACAGAAAAATCTAACCAACTTTACAC
TACCACTTCACATCAGACATCTTTAGTAAGGAATAGTGCATCACTTTATTGCATGCTTCCTTGGCATCATGTCAA
TAGATTTAACTTTGTATTTAGTTCCACAGGATGCAAGATCAGTATAGAATATATTTTAAAAGATCTTAAGATTAA
AGATCCCAGTTGTATAGCATTCATAGGTGAAGGAGCTGGTAACTTATTATTACGTACGGTAGTAGAACTTCATC
CTGACATAAGATATATTTACAGAAGTTTAAAAGATTGCAATGATCATAGTTTACCTATTGAATTTCTAAGGTTAT
ACAACGGGCATATAAACATAGATTATGGTGAGAATTTAACCATTCCTGCTACAGATGCAACTAACAACATACAT
TGGTCTTATTTACACATAAAATTTGCAGAACCTATTAGTATCTTTGTCTGTGATGCTGAATTACCTGTCACAGCC
AATTGGAGTAAAATCATAATTGAATGGAGTAAGCATGTAAGAAAGTGCAAATACTGTTCTTCTGTAAATAGAT
GCATTTTAATTGCAAAATACCATGCTCAAGATGATATTGATTTCAAATTAGATAACATTACTATATTGAAAACTT
ATGTGTGCCTAGGTAGCAAGTTAAAAGGATCTGAAGTTTACTTAGTCCTTACAATAGGCCCTGCAAATATACT
TCCTGTTTTTGATGTTGTGCAAAATGCTAAATTGATTCTTTCAAGAACTAAAAATTTCATTATGCCTAAAAAGA
TTGACAAGGAATCTATCGATGCAAATATTAAAAGCTTAATACCTTTCCTTTGTTACCCTATAACAAAAAATGGA
ATTAAGACTTCATTGTCAAAATTGAAGAGTGTAGTTAATGGAGATATATTATCATATTCTATAGCTGGACGTAAT
GAAGTATTCAGCAACAAGCTTATAAACCACAAGCATATGAATATCTTAAAATGGCTGGATCATGTTTTAAACTT
TAGATCAGCTGAACTTAATTACAATCATTTATACATGATAGAGTCCACATATCCTTACTTGAGTGAATTATTAAAT
AGTTTAACAACCAATGAGCTCAAGAAGCTGATTAAAATAACAGGTAGTGTACTATACAACCTTCCTAATGAAC
AGTAACTTAAAATATCATTAACAAGTTTGGTCAAATTTAGATGCTAACACATTATTATATTATAGTTATTAAAAA
AATATGCAAACTTTTCAATAATTTAGCATATTGATTCCAAGATTATCATTTTAGTCTTAAGGGATTAAATAAAAG
TCTAAAACTAACAATCACACATGTGCATTTACAACAC

>Sequence-rRSV BA9 Δ7AA

TGCGAAAAAAAATGCGTACTACAAACTTGCACACTCGAAAAAAATGGGGCAAATAAGAATTTGATAAGTGCT
ATTTAAGTCTAACCTTTTTAATCAGAAATGGGGTGCAATTCACTGAGCATGATAAAGGTTAGATTACAAAATT
TGTTTGATAATGACGAAGTAGCATTGTTAAAAATAACATGTTATACTGACAAATTAATTCTTCTGACTAATGCAT
TAGCCAAAGCAACAATACATACAATTAAATTAAACGGCATAGTTTTTATACATGTTATAACAAGCAGTGAAGTG
TGCCCTGATAACAATATTGTAGTGAAATCTAACTTTACAACAATGCCAATATTACAAAATGGAGGATACATATG
GGAATTGATTGAATTGACACACTGCTCTCAATTAAATGGTCTAATAGATGATAATTGTGAAATCAAATTTTCTA
AAAGACTAAGTGACTCAGTAATGACTGATTATATGAATCAAATATCTGATTTACTTGGGCTTGATCTCCATTCAT
GAATTATGTTTAGTCTAATTCAATAGACATGTGTTTATTACCATTTTAGTTAATATAAAACCTCATCAAAGGGAA
ATGGGGCAAATAAACTCACCCAATCAATCAAACCATGAGCACTACAAACGACAACACCACCATGCAAAGATT
GATGATCACAGACATGAGACCCCTGTCGATGGATTCAATAATAACATCTCTCACTAAAGAAATCATCACACAC
AAATTCATATACTTGATAAACAATGAATGTATTGTAAGAAAACTCGATGAAAGACAAGCTACATTTACATTCCT
AGTCAATTATGAGATGAAGCTATTGCACAAAGTAGGGAGCACCAAATACAAGAAATACACCGAATATAATAC
AAAATATGGCACATTCCCTATGCCTATATTTATCAATCATGGCGGGTTTCTAGAATGTATTGGCATTAAGCCTAC
AAAACATACTCCTATAATATACAAATATGACCTCAACCCGTAACTTCCAACAAAAAAACCAACTCATCCAAACC

AAGCCATTCTCCAAACAACAATGCTCAACAGTTAAGAAGGAGCTAATCCATTTTAGTAATTAAAAATAAGGGT
GAAACCAGTAACATAAATTGGGGCAAATACAAAGATGGCTCTTAGCAAAGTCAAGTTGAATGATACATTAAA
TAAGGATCAGCTGCTGTCATCCAGCAAATACACTATTCAACGTAGTACAGGAGATAATATTGACACTCCCAATT
ATGATGTGCAAAAACACCTAAACAAACTATGTGGTATGCTATTAATCACTGAAGATGCAAATCATAAATTCACA
GGATTAATAGGTATGCTATATGCTATGTCCAGATTAGGAAGAGAAGACACTATAAAGATACTTAAAGATGCTG
GATATCATGTTAAAGCTAATGGAGTAGATATAACAACATATCGTCAAGATATAAATGGAAAGGAAATGAAATTC
GAAGTATTAACATTATCAAGCTTGACATCAGAAATACAAGTCAATATTGAGATAGAATCTAGAAAGTCCTACA
AAAAAATGCTAAAAGAGATGGGAGAAGTGGCTCCAGAATATAGGCATGATTCTCCAGACTGTGGGATGATA
ATACTGTGTATAGCTGCCCTTGTAATAACCAAATTAGCAGCAGGAGATAGATCAGGTCTTACAGCAGTAATTA
GGAGGGCAAACAATGTCTTAAAAAACGAAATAAAACGCTACAAGGGCCTAATACCAAAAGACATAGCCAAC
AGTTTTTATGAAGTGTTTGAAAAATACCCTCATCTTATAGATGTTTTTGTGCACTTTGGCATAGCACAATCATC
CACAAGAGGGGGCAGTAGAGTTGAAGGAATCTTTGCAGGATTGTTTATGAATGCCTATGGTTCAGGACAAG
TAATGCTAAGATGGGGAGTTTTAGCCAAATCTGTAAAAAATATCATGCTAGGACATGCTAGTGTCCAAGCAGA
AATGGAGCAAGTTGTGGAAGTCTATGAGTATGCACAGAAGTTGGGAGGAGAAGCTGGTTTCTACCATGTAT
TGAACAATCCAAAAGCATCATTGCTGTCATTAACTCAATTTCCTAACTTCTCAAGTGTGGTCCTAGGCAATGC
AGCAGGTCTAGGCATAATGGGAGAGTATAGAGGTACACCAAGAAACCAAGATCTCTATGATGCAGCCAAAG
CATATGCAGAGCAACTCAAAGAAAATGGAGTAATAAACTACAGTGTATTAGACTTAACAACAGAAGAATTGG
AAGCCATAAAGCATCAACTCAACCCCAAAGAAGATGACGTAGAGCTTTAAGTTAACAAAAAATACGGGGCA
AATAAGTCAACATGGAGAAGTTTGCACCTGAATTTCATGGAGAAGATGCAAATAACAAAGCTACCAAATTCC
TAGAATCAATAAAAGGCAAGTTTGCATCATCCAAAGATCCTAAGAAGAAAGATAGCATAATATCTGTCAACTC
AATAGACATAGAAGTCACTAAAGAGAGCCCGATAACATCTGGCACCAACATTATCAATCCAACAAGTGAAGC
CGACAGTACCCCAGAAACTAAAGCCAACTACCCAAGAAAACCCCTAGTAAGCTTCAAAGAAGATCTCACCC
CAAGTGATAACCCTTTCTCTAAGTTGTACAAAGAAACCATAGAAACATTTGATAACAATGAAGAAGAATCTAG
CTACTCATATGAGGAGATCAATGACCAAACAAATGACAACATTACAGCAAGACTAGATAGAATTGATGAAAA
ATTAAGTGAAATATTAGGAATGCTCCATACATTAGTAGTTGCAAGTGCAGGACCTACTTCGGCTCGTGACGGA
ATAAGAGATGCTATGGTTGGTCTAAGAGAAGAAATGATAGAAAAAATAAGAGCAGAAGCATTAATGACCAAT
GATAGGTTAGAGGCTATGGCAAGACTTAGGAATGAGGAAAGCGAAAAAATGGCAAAAGACACCTCAGATG
AAGTGTCTCTCAATCCAACCTCTAAAAAATTGAGTGACTTGTTGGAAGACAACGATAGCGACAATGATCTATC
ACTTGATGATTTTTGATCAGTGATCAACTCACTCAGCAATCAACAACATCAATAAAACAGACACCAATCCATT
GAATCAATTGCCAGACTGAAAAAACAAACATCCATCAGCAGAACCACCAACCAATCAATCAACCAATTGATC
AATCAGCACCCTGACAAAATTAACAATATAGTAACAAAAAAAGAACAAGATGGGGCAAATATGGAAACATAC
GTGAACAAGCTTCACGAAGGCTCCACATACACAGCAGCTGTCCAGTACAATGTTCTAGAAAAAGATGATGAT
CCCGCATCACTAACAATATGGGTGCCTATGTTCCAGTCATCTGTGCCAGCAGACTTGCTCATAAAAGAACTTG
CAAGCATCAACATACTAGTAAAGCAGATCTCTACGCCCAAAGGACCTTCACTACGAGTCACGATCAACTCAA
GAAGTGCTGTGCTGGCTCAAATGCCTAGTAATTTCACCATAAGTGCAAATGTATCATTAGATGAAAGAAGCAA
ATTAGCATATGATGTAACTACACCTTGTGAAATCAAAGCATGCAGTTTAACATGCTTAAAAGTAAAAAGTATGT
TAACTACAGTCAAAGATCTAACCATGAAGACATTCAACCCCACTCATGAGATCATTGCTCTATGTGAATTTGAA
AATATTATGACATCAAAAAGAGTAATAATACCAACCTATCTAAGATCAATCAGTGTCAAGAACAAAGATCTGA
ACTCACTGGAAAATATAGCAACCACCGAATTCAAAAATGCTATCACCAATGCTAAAATTATTCCCTATGCAGGA
TTAGTGTTAGTTATCACGGTTACTGATAATAAAGGAGCATTCAAGTATATCAAGCCACAGAGTCAATTTATAGT
GGATCTTGGTGCCTACCTAGAAAAAGAGAGCATATATTATGTGACTACTAATTGGAAGCATACAGCTACACGT
TTTTCAATCAAACCACTAGAGGATTAAACTCAATTATCAACATTGAATGACAGGTTCACATACATCCTCAACTG
CACACTGTATCTAAACATCATAAACATCTACACTACACACTTCATCACACAAACCAATCCCACTCAAAATCTAA
AATCACTTCCAGCCATTGTCTGCCAGACCTAGAGTGCGAATAGGTATATAAAACAAGAATATGGGGTAAATAG

ATATCAGTTAGAGTTCAACCAATCTCAACAACCATCTATACCGCCAATCCAATACATACATTGCAAATCTTAAAA
TGGGAAACACATCCATCACAATAGAATTCACAAGCAAATTTTGGCCTTATTTTACACTAATACACATGATCTTA
ACTCTAATCTCTTTACTAATTATAATCACTATTATGATTGCAATACTAAATAAGCTGAGTGAACATAAAATATTCT
GCAACAAAACTCTTGAGCAAGGACAGATGTATCAAATCGACACATAGTGTTCTCCCATTATGCTGTATCAAAT
CACAATCCTGTGTATATAAATAAACAAATCCAATCTTCTCACAGAGTCATGGCATCACAAAACCATGCCAACCA
TCATGGTAGCATAGAGTAGTTATTAAAAATTAACATAATGATGAATTATGAGTATGGGATCAAAAACAACATTG
GGGCAAATGCAACAATGTCCAAAAACAAGAATCAACGCACTGCCAGGACTCTAGAAAAGACCTGGGATACT
CTTAATCATCTAATTGTAATATCCTCTTGTTTATACAAATTAAATTTAAAATCTATAGCACAAATAGCACTATCAG
TTTTGGCAATGATAATCTCAACCTCTCTTATAATTGCAGCCATAATATTCATCATCTCTGCCAATCACAAAGTTA
CACTAACAACTGTCACAGTTCAAACAATAAAAAACCACACTGAGAAAAACATAACCACTTACCTTACTCAAG
TCTCGCCAGAAAGGGTTAGCCCATCCAAACAACCCACAGCCACACCGCCAATCCACACAAACTCAGCCACA
ATATCACCCAATACAAAATCAGATACACACCATACAACAACACAAACCAAAGGCACAATCTCTACTCCAACAC
AGAACAACAAGCCAAGCACAAAACCACGTCCAAAAAATCCACCAAAAAAAGATGATTACCATTTTGAAGTG
TTCAACTTTGTTCCCTGTAGTATATGTGGCAGCAATCAACTCTGCAAATCCATTTGCAAAACAATACCAAGCAA
TAAACCAAAGAAAAAACCAACCACAAAACCCACAAACAAACCACCTACCAAAACCACAAACAAAAGAGAC
CCCAAAACACTAGCCAAAACACCGAAAAAAGAAACCACCATTAACCCAACAAAAAAACCAACCCCCAAGAC
TACAGAAAGAGACACCAGCACACCACAATCCACTGTGCTCGACACAACCACATCAAAACACACAGAAAGG
GACACCAGCACCTCACAATCCATTGTGCTTGACACAACCGCATCAAAACACACAACCCAACAGCAATCTCTC
TACTCAACCATCCCTGAAAACACACCCAACTCCACACAAACACCCACAGCATCTGAGCCCTCTACATCAAATT
CTATCTAGTTATTTAAAAACTACATCTTAGCAAAGAACCGTGATCCCTTAAGCAAGAACGAAATTAAATCTGG
GGCAAATAACCATGGAGTTGCTGATCCATAGATCAAGTGCAATCTTCCTAACTCTTGCTATTAATGCATTGTAC
CTTACCTCAAGTCAGAACATAACTGAGGAGTTTTACCAATCAACATGTAGTGCAGTTAGCAGAGGTTACTTG
AGTGCTTTAAGAACAGGTTGGTATACCAGTGTCATAACAATAGAATTAAGTAATATAAAAGAAACCAAATGCA
ATGGAACTGACACTAAAGTTAAACTTATAAAACAAGAATTAGATAAGTATAAGAATGCAGTAACTGAATTACA
GTTACTTATGCAAAACACACCAGCTGTCAACAACCGGGCCAGAAGAGAAGCACCACAGTATATGAACTACA
CAATCAATACCACTAAAAACCTAAATGTATCAATAAGCAAGAAGAGGAAACGAAGATTTCTGGGCTTCTTGTT
AGGTGTAGGATCTGCAATAGCAAGTGGTATAGCTGTATCCAAAGTTCTACACCTTGAAGGAGAAGTGAACAA
GATCAAAAATGCTTTGCAGCTTACAAACAAAGCTGTAGTCAGTCTATCAAATGGGGTCAGTGTTTTAACCAG
CAGAGTGTTAGATCTCAAGAATTATATAAACAACCAATTATTACCTATGGTAAATCGACAGAGTTGTCGCATAT
CCAACATTGAGACAGTTATAGAATTCCAGCAGAAGAACAGCAGATTGTTGGAAATCACCAGAGAATTTAGT
GTCAATGCAGGTGTAACGACACCTTTAAGCACTTATATGTTAACAAACAGTGAGTTACTATCATTAATCAATGA
TATGCCTATAACAAATGATCAGAAAAAATTAATGTCAAGCAATGTTCAGATAGTAAGGCAACAAAGTTATTCTA
TCATGTCTATAATAAAGGAAGAAGTCCTTGCATATGTTGTACAGCTACCTATCTATGGTGTAATTGATACACCTT
GCTGGAAATTACACACATCACCTCTGTGCACCACCAACATCAAAGAAGGATCAAATATTTGTTTAACAAGGA
CTGATAGAGGATGGTATTGTGATAATGCAGGATCAGTATCCTTCTTTCCACAAGCTGACACTTGTAAAGTACA
GTCCAATCGAGTATTTTGTGACACTATGAACAGTTTGACATTACCAAGTGAAGTCAGCCTTTGTAACACTGAC
ATATTCAATTCCAAGTATGACTGCAAAATTATGACATCAAAAACAGACATAAGCAGCTCAGTAATTACTTCTCT
AGGAGCTATAGTGTCATGCTATGGCAAGACTAAATGTACTGCATCCAACAAAAATCGTGGAATTATAAAGACA
TTTTCTAATGGTTGTGATTATGTGTCAAACAAAGGAGTAGATACTGTATCAGTGGGCAACACTTTATATTATGT
CAACAAGCTGGAAGGCAAAAACCTTTATGTAAAAGGGGAACCTATAATAAATTACTATGACCCTCTAGTGTTT
CCTTCTGATGAGTTTGATGCATCAATATCTCAAGTCAATGAAAAAATTAATCAAAGTTTAGCTTTTATTCATAGA
TCTGATGAATTATTACATAATGTAAATACTGGAAAATCTACTACAAATATTATGATAACTGCAATTATTATAGTAA
TCATTGTAGTATTGTTATCATTAATAGCTATTGGTTTACTGTTGTATTGCAAAGCCAAAAACACACCAGTTACAT
TAAGCAAAGACCAACTAAGTGGAATCAATAATATTGCATTCAGCAAATAGACAAAAAACCACCTGATCATGTT

CCAACAACAATCTGCTGACCATCAATCCCAAATCAACTTACAACAGATACTTCAACATCACAGTACAGGCTGA
ATCATCTCCTCGCATCATGCTACCTACACAACTAAGCTAGATCCTTAATTCATAGTTACATAAAAGCCTCAAATA
TCGCAATCAACACTAAATCAACACATCATTTACAAAACTAACAGCTGGGGCAAATATGTCGCGAAGAAATCCC
TGCAAATTTGAGATTAGAGGTCATTGCTTGAATGGTAGAAGATGCCACTACAGTCATAATTACTTTGAATGGC
CTCCTCATGCATTGCTAGTGAGGCAAAACTTCATGTTAAACAAGATACTCAAGTCAATGGACAAGAGCATAG
ACACTTTGTCTGAAATAAGTGGAGCTGCTGAACTTGATAGAACAGAAGAATATGCTCTTGGTATAGTTGGAG
TGCTAGAGAGTTACATAGGATCTATAAACAACATAACAAAACAATCAGCATGTGTTGCTATGAGTAAACTTCTT
ATTGAGATCAATAGTGATGACATTAAAAAGCTGAGAGATAATGAAGAACCCAATTCACCTAAGATAAGAGTG
TACAATACTGTTATATCATACATCGAGAGCAATAGAAAAAACAGCAAGCAAACCATCCATCTGCTTAAACGATT
ACCAGCAGACGTGCTGAAGAAGACAATAAAGAACACATTAGATATCCACAAAAGCATAACCATAAGCAACCC
AAAAGAGTCAACCATAAGTGATCAAAATGACCAAACCAAAAATAATGATATTACCGGATAAATATCCTTGTAG
TATATCATCCATATTGATCTCAAGTGAAAGCATGATTGCTACATTCAACCATAAAGACATATTACAATTTAACCA
CAACCATTTAGATAACCACCAGTGTTTATTAAATCATATATTTGATGAAATTCATTGGACACCTAAAAACTTATT
AGATGCCACTCAACAATTTCTCCAACATCTTAACATCCCTGAAGATATATATACAGTATATATATTAGTGTCATAA
TGCTTGATCATAACGATTCTATATCATCCAACCATAAAATTGTCTTAATAAAGTCATGGGACAAAATGGATCCCA
TTATTAATGGTAGCTCTGCTAATGTATATCTAACTGATAGTTATCTAAAAGGTGTTATCTCTTTTTCAGAATGTAA
TGCTTTAGGGAGTTACCTTTTTAACGGCCCTTATCTTAAAAATGATTATACCAACTTAATTAGTAGACAAAGCC
CACTAATAGAGCATATGAATCTAAAAAAACTAACTATAACACAGTCATTAATATCTAGATACCATAAAGGTGAA
CTGAAATTAGAAGAACCAACTTATTTCCAGTCATTACTTATGACATATAAAAGCATGTCCTCGTCTGAACAAAT
TGCTACAACTAACTTACTTAAAAAAATAATACGAAGAGCTATAGAAATAAGTGATGTAAAGGTGTACGCCATC
TTGAATAAACTAGGACTAAAGGAAAAGGACAGAGTTAAGCCCAACAACAATTCAGGTGATGAAAACTCAGT
ACTTACAACCATAATTAAAGATGATATACTCTCAGCTGTGGAAAACAATCAATCATATACAAATTCAGATAAAA
ATTACTCAGTAAATCAAAATATCAATATCAAAACAACACTCTTAAAAAAGTTGATGTGTTCAATGCAACATCCT
CCATCATGGTTAATACACTGGTTCAATTTATATACAAAATTAAATAACATATTAACACAATATCGATCAAATGAG
GTAAAAAGTCATGGGTTTATATTAATAGATAATCAAACTTTGAGTGGTTTTCAGTTTATTTTAAATCAATATGGT
TGCATTGTTTATCATAAAGGGCTCAAAAAAATTACAACTACTACATACAATCAATTTTTGACATGGAAAGACAT
CAGCCTCAGCAGATTAAATGTTTGCTTAATTACTTGGATAAGTAATTGTTTAAATACATTAAATAAAAGCTTAG
GGTTGAGATGCGGATTCAATAATGTTGTGCTATCACAATTATTCCTTTACGGAGATTGTATACTGAAATTATTTC
ATAATGAAGGCTTTTACATAATAAAAGAAGTAGAAGGATTTATTATGTCTTTAATTCTAAACATAACAGAAGAA
GATCAATTTAGGAAACGATTTTATAATAGCATGCTAAATAACATCACAGATGCAGCTATTAAGGCTCAAAAGG
ATCTACTATCAAGAGTATGTCACACTTTATTAGACAAGACAGTGTCTGATAATATCATAAATGGTAAATGGATAA
TTCTATTAAGTAAATTTCTTAAATTGATTAAGCTTGCAGGTGATAATAATCTCAATAACTTGAGTGAGCTATATT
TTCTCTTCAGAATCTTTGGACATCCAATGGTTGATGAGAGACAAGCAATGGATGCTGTAAGAATTAACTGCAA
TGAAACTAAGTTCTATTTATTAAGTAGCCTAAGTACGTTGAGAGGTGCTTTCATTTATAGAATCATAAAAGGGT
TTGTAAATACCTACAACAGATGGCCCACTTTAAGGAATGCTATTGTCCTACCTCTAAGATGGTTAAACTATTATA
AACTTAATACTTATCCATCTCTACTTGAAATCACAGAAAATGATTTGATTATTTTATCAGGATTGCGGTTCTATC
GTGAGTTTCATCTGCCTAAAAAAGTGGATCTTGAAATGATAATAAATGACAAAGCTATTTCTCCTCCAAAAGA
TCTAATATGGACTAGTTTTCCTAGAAATTACATGCCATCACATATACAAAATTATATAGAACATGAAAAGTTGAA
GTTCTCTGAAAGCGACAGATCAAGAAGAGTACTAGAGTATTACTTGAGAGATAATAAATTCAATGAATGTGAT
CTATACAATTGTGTAGTTAATCAAAGCTATCTCAACAACTCTAATCATGTGGTATCACTAACTGGTAAAGAAAG
AGAGCTCAGTGTGGGTAGAATGTTTGCTATGCAACCAGGTATGTTTAGGCAAATCCAAATCTTAGCAGAAAA
AATGATAGCCGAAAATATTTTACAATTCTTCCCTGAGAGTTTGACAAGATATGGTGATCTAGAGCTTCAAAAG
ATATTAGAATTAAAAGCAGGAATAAGCAACAAGTCAAATCGTTATAATGATAACTACAACAATTATATCAGTAA
ATGTTCTATAATAACAGATCTTAGCAAATTTAATCAAGCATTTAGATATGAAACATCATGTGTCTGCAGTGATGT

ATTAGATGAACTGCATGGGGTACAATCTCTATTCTCTTGGTTGCATTTAACAATACCTCTTGTCACAATAATATG
TACATATAGACATGCACCTCCTTTTATAAAGGATCATGTTGTCAATCTTAATGAAGTTGATGAACAAAGTGGGT
TATACAGATATCATATGGGTGGTATTGAGGGCTGGTGTCAAAAACTGTGGACCATTGAAGCCATATCATTATTA
GATCTAATATCTCTTAAAGGTAAATTCTCCATCACAGCTCTGATAAATGGTGATAATCAGTCAATTGATATAAGT
AAACCAGTTAGACTTATAGAGGGTCAGACCCATGCTCAAGCAGATTATTTGTTAGCATTAAATAGCCTTAAGT
TGCTATATAAAGAGTATGCAGGCATAGGCCATAAGCTTAAGGGAACTGAGACCTATATATCTAGAGATATGCA
GTTCATGAGCAAAACAATCCAGCACAATGGAGTGTACTATCCAGCCAGTATCAAAAAAGTCCTGAGAGTAGG
TCCATGGATAAATACAATACTTGATGATTTTAAAGTCAGTTTAGAATCTATAGGTAGCTTAACACAGGAGTTAG
AATACAGAGGAGAAAGCTTATTATGCAGTTTAATATTTAGGAATATTTGGTTATACAATCAAATTGCTCTGCAA
CTCCGAAATCATGCTTTATGTAATAATAAGCTATATTTAGATATATTGAAGGTATTAAAACACTTAAAAACCTTT
TTTAATCTTGATAGTATCGATACGGCGTTATCATTGTATATGAACTTGCCTATGCTGTTTGGTGGTGGTGATCCT
AATTTGTTATATCGAAGCTTTTATAGGAGAACTCCAGACTTCCTTACAGAAGCTATAGTACATTCAGTGTTTGT
GTTGAGCTATTATACTGGTCACGATCTACAAGATAAGCTCCAGGATCTTCCAGATGATAGACTGAACAAATTC
TTGACTTGTGTCATCACATTTGATAAAAATCCAAATGCCGAGTTTGTAACATTGATGAGGGATCCACAGGCTT
TAGGGTCTGAAAGGCAAGCTAAAATTACTAGTGAGATTAATAGATTAGCAGTAACAGAAGTCTTAAGTATAG
CTCCAAACAAAATATTTTCTAAAAGTGCGCAACACTATACTACCACTGAGATTGATCTAAATGACATTATGCAA
AATATAGAACCAACTTACCCTCATGGATTAAGAGTTGTTTATGAAAGTTTACCTTTTTATAAAGCAGAAAAAAT
AGTTAATCTTATATCAGGAACAAAATCCATAACTAATATACTTGAAAAAACATCAGCGATAGATACAACTGATA
TTAATAGGGCTACTGATATGATGAGGAAAAATATAACCTTACTTATAAGGATACTTCCACTAGATTGTAACAAA
GACAAAAGAGAGTTATTAAGTTTAGAAAATCTTAGCATAACTGAATTAAGCAAGTATGTAAGAGAAAGATCT
TGGTCATTATCCAATATAGTAGGAGTAACATCACCAAGTATTATGTTCACAATGGACATTAAATATACAACTAGC
ACTATAGCCAGTGGTATAATTATAGAAAAATATAATGTTAATGGTTTAACTCGTGGTGAAAGAGGACCTACTAA
ACCATGGGTAGGTTCATCTACACAGGAGAAAAAAACAATGCCAGTGTACAATAGACAAGTTTTAACCAAAA
AGCAAAGAGACCAAATAGATTTATTAGCAAAATTAGACTGGGTATATGCATCCATAGACAACAAAGATGAATT
CATGGAAGAACTGAGTACTGGAACACTTGGACTGTCATATGAAAAGGCCAAAAAGTTGTTTCCACAATATCT
AAGTGTCAATTATTTACACCGGTTAACAGTCAGTAGTAGACCATGCGAATTCCCTGCCTCAATACCAGCTTATA
GAACAACAAATTATCATTTTGATACTAGTCCTATCAATCATGTATTAACAGAAAAGTATGGAGATGAAGATATC
GACATTGTGTTTCAAAATTGCATAAGTTTTGGTCTTAGCTTGATGTCAGTTGTGGAACAATTCACAAACATAT
GTCCTAATAGAATTATTCTCATACCGAAGCTGAATGAGATACATTTGATGAAGCCTCCTATATTTACAGGAGAT
GTTGATATCATCAAGTTGAAGCAAGTGATACAAAAACAGCATATGTTCCTACCAGATAAAATAAGTTTAACCC
AATATGTAGAATTATTCTTAAGTAACAAAGCACTTAAATCTGGATCTCACATCAACTCTAATTTAATATTAGCAC
ATAAAATGTCTGATTATTTTCATAATGCGTATATTTTAAGTACTAATTTAGCTGGTCATTGGATTCTGATTATTCA
ACTTATGAAAGATTCAAAAGGTATTTTTGAAAAAGATTGGGGAGAGGGGTATATAACTGATCATATGTTCATT
AATTTGAATGTTTTCTTTAATGCTTATAAGACTTATTTGCTATGTTTTCATAGAGGTTATGGTAAAGCAAAATTA
GAATGTGATATGAACACTTCAGATCTTCTTTGTGTTTTGGAGTTAATAGACAGTAGCTACTGGAAATCTATGTC
TAAAGTTTTCCTAGAACAAAAAGTCATAAAATACATAGTCAATCAAGACACAAGTTTGCATAGAATAAAAGGT
TGTCACAGTTTTAAGTTGTGGTTTTTAAAACGCCTTAATAATGCTAAATTTACCGTATGCCCTTGGGTTGTTAA
CATAGATTATCACCCAACACACATGAAAGCTATATTATCTTACATAGATTTAGTTAGAATGGGGTTAATAAATGT
AGATAAATTAACCATTAAAAATAAAAACAAATTCAATGATGAATTTTACACATCAAATCTCTTTTACATTAGTTA
CAACTTTTCAGACAACACTCATCTGCTAACAAAACAAATAAGGATTGCTAATTCAGAATTAAAAGATAATTATA
ACAAACTATATAACCCAACCCCAGAAGCTTTAGAAAATGTATCATTAATCCCTGTTAAAAGTAATAATAGAAAC
AAACCTAAATTTTGTATAAGTGGAAGTACTGAATCTATGATGACGTCAACATTCTCTAATAAAATGCATATTAAA
TCTTCCACTGTTACCACAAGATTCAATTATAGCAGACAAGACTTGTACAATTTATTTCCAATTGTTGTGATAGA
CAGGATTATAGATCATTCAGGTAATACAGAAAAATCTAACCAACTTTACACTACCACTTCACATCAGACATCTT

TAGTAAGGAATAGTGCATCACTTTATTGCATGCTTCCTTGGCATCATGTCAATAGATTTAACTTTGTATTTAGTT
CCACAGGATGCAAGATCAGTATAGAATATATTTTAAAAGATCTTAAGATTAAAGATCCCAGTTGTATAGCATTC
ATAGGTGAAGGAGCTGGTAACTTATTATTACGTACGGTAGTAGAACTTCATCCTGACATAAGATATATTTACAG
AAGTTTAAAAGATTGCAATGATCATAGTTTACCTATTGAATTTCTAAGGTTATACAACGGGCATATAAACATAG
ATTATGGTGAGAATTTAACCATTCCTGCTACAGATGCAACTAACAACATACATTGGTCTTATTTACACATAAAAT
TTGCAGAACCTATTAGTATCTTTGTCTGTGATGCTGAATTACCTGTCACAGCCAATTGGAGTAAAATCATAATT
GAATGGAGTAAGCATGTAAGAAAGTGCAAATACTGTTCTTCTGTAAATAGATGCATTTTAATTGCAAAATACC
ATGCTCAAGATGATATTGATTTCAAATTAGATAACATTACTATATTGAAAACTTATGTGTGCCTAGGTAGCAAGT
TAAAAGGATCTGAAGTTTACTTAGTCCTTACAATAGGCCCTGCAAATATACTTCCTGTTTTTGATGTTGTGCAA
AATGCTAAATTGATTCTTTCAAGAACTAAAAATTTCATTATGCCTAAAAAGATTGACAAGGAATCTATCGATGC
AAATATTAAAAGCTTAATACCTTTCCTTTGTTACCCTATAACAAAAAATGGAATTAAGACTTCATTGTCAAAATT
GAAGAGTGTAGTTAATGGAGATATATTATCATATTCTATAGCTGGACGTAATGAAGTATTCAGCAACAAGCTTA
TAAACCACAAGCATATGAATATCTTAAAATGGCTGGATCATGTTTTAAACTTTAGATCAGCTGAACTTAATTAC
AATCATTTATACATGATAGAGTCCACATATCCTTACTTGAGTGAATTATTAAATAGTTTAACAACCAATGAGCTC
AAGAAGCTGATTAAAATAACAGGTAGTGTACTATACAACCTTCCTAATGAACAGTAACTTAAAATATCATTAAC
AAGTTTGGTCAAATTTAGATGCTAACACATTATTATATTATAGTTATTAAAAAAATATGCAAACTTTTCAATAATT
TAGCATATTGATTCCAAGATTATCATTTTAGTCTTAAGGGATTAAATAAAAGTCTAAAACTAACAATCACACAT
GTGCATTTACAACAC

NOTE:

The nucleic acid mutation site (A8580T) of the rescue strain is a silencing mutation specifically designed to serve as a molecular marker to distinguish it from the parental strain.
